# Laser-activatable oxygen self-supplying nanoplatform for efficiently overcoming colorectal cancer resistance by enhanced ferroptosis and alleviated hypoxic microenvironment

**DOI:** 10.1186/s40824-023-00427-1

**Published:** 2023-09-23

**Authors:** Hao Jiang, Hailong Tian, Zhihan Wang, Bowen Li, Rui Chen, Kangjia Luo, Shuaijun Lu, Edouard C. Nice, Wei Zhang, Canhua Huang, Yuping Zhou, Shaojiang Zheng, Feng Gao

**Affiliations:** 1 The First Hospital of Ningbo University, Ningbo, 315020 China; 2 State Key Laboratory of Biotherapy and Cancer Center, West China School of Basic Medical Sciences & Forensic Medicine, Collaborative Innovation Center for Biotherapy, West China Hospital, Sichuan University, Chengdu, 610041 China; 3 Department of Biochemistry and Molecular Biology, Monash University, Clayton, VIC 3800 Australia; 4 Hainan Cancer Center and Tumor Institute, The First Affiliated Hospital of Hainan Medical University, Haikou, 570102 China

**Keywords:** Colorectal cancer, Chemo-resistance, Ferroptosis, Chemo-photothermal therapy, Hypoxia

## Abstract

**Background:**

Colorectal cancer (CRC) is the second most deadly cancer worldwide, with chemo-resistance remaining a major obstacle in CRC treatment. Notably, the imbalance of redox homeostasis-mediated ferroptosis and the modulation of hypoxic tumor microenvironment are regarded as new entry points for overcoming the chemo-resistance of CRC.

**Methods:**

Inspired by this, we rationally designed a light-activatable oxygen self-supplying chemo-photothermal nanoplatform by co-assembling cisplatin (CDDP) and linoleic acid (LA)-tailored IR820 via enhanced ferroptosis against colorectal cancer chemo-resistance. In this nanoplatform, CDDP can produce hydrogen peroxide in CRC cells through a series of enzymatic reactions and subsequently release oxygen under laser-triggered photothermal to alleviate hypoxia. Additionally, the introduced LA can add exogenous unsaturated fatty acids into CRC cells, triggering ferroptosis via oxidative stress-related peroxidized lipid accumulation. Meanwhile, photothermal can efficiently boost the rate of enzymatic response and local blood flow, hence increasing the oxygen supply and oxidizing LA for enhanced ferroptosis.

**Results:**

This nanoplatform exhibited excellent anti-tumor efficacy in chemo-resistant cell lines and showed potent inhibitory capability in nude mice xenograft models.

**Conclusions:**

Taken together, this nanoplatform provides a promising paradigm via enhanced ferroptosis and alleviated hypoxia tumor microenvironment against CRC chemo-resistance.

**Graphical Abstract:**

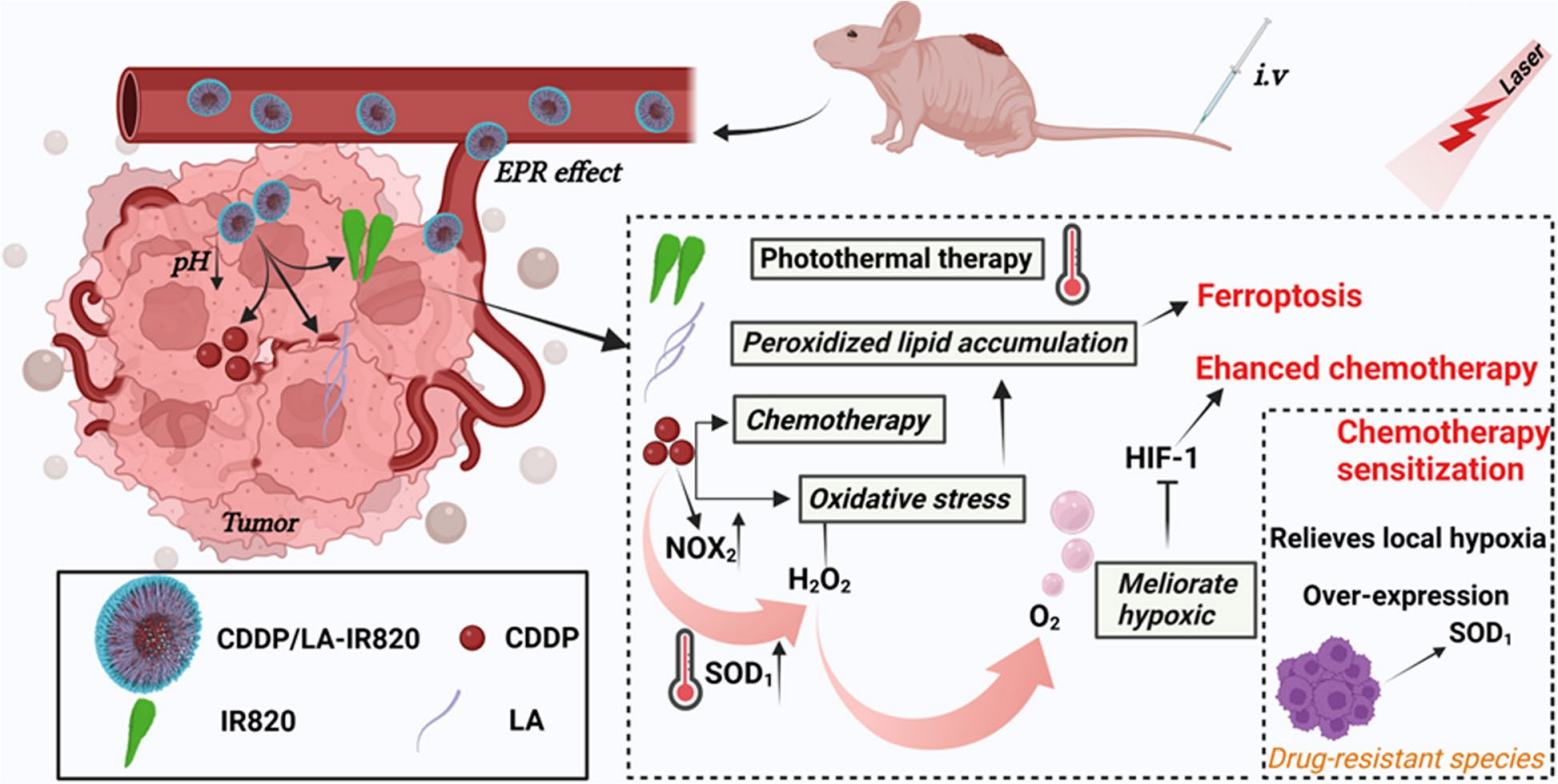

**Supplementary Information:**

The online version contains supplementary material available at 10.1186/s40824-023-00427-1.

## Introduction

Colorectal cancer (CRC) is one of the most common malignant tumors that seriously endanger human health because of its low cure rate [1]. Moreover, chemotherapy resistance is one of the main causes of recurrence in CRC patients and leads to poor prognosis. Emerging evidence has demonstrated that the hypoxic microenvironment commonly exists in solid tumors, and is tightly associated with cancer progression and resistance to therapy [2]. Particularly, large amounts of oxygen are consumed during the generation of drug-induced reactive oxygen species (ROS), which frequently leads to a poor response to pro-oxidative stress therapy. Therefore, relieving CRC tumor hypoxia is expected to be an effective way to enhance the anti-tumor effect and reverse resistance to pro-oxidative therapeutic agents [3].

Notably, another key pathophysiological feature of drug-resistant cancer cells that might be exploited is that they live in redox homeostasis, which is dynamically balanced at a level much higher than drug-sensitive cancer cells. Cellular redox homeostasis, a dynamic balance between the generation and elimination of ROS, is fundamentally important for maintaining a physiological steady state within a living cell. Unsurprisingly, drug-resistant cells have been shown to have high levels of antioxidant enzymes and other factors responsible, such as superoxide dismutase (SOD) and glutathione (GSH), for the production of antioxidants, which corresponds to chemotherapy resistance [4]. Consequently, drug-resistant cancer cells may be more susceptible to changing ROS levels, and utilizing this vulnerability to enhance chemotherapeutic response is expected. It should be noted that ferroptosis, a novel form of cell death associated with oxidative stress, can be used as an alternative pathway to overcome the resistance of conventional chemotherapy. Several studies have explored the manipulation of redox homeostasis in cancer cells by releasing Fe ions from nanoparticles to trigger ferroptosis. In these studies, stimuli-responsive nanoparticles were designed to release Fe ions into the tumor microenvironment, thereby disrupting redox balance and inducing ferroptosis [5–7]. On the other hand, under oxidative stress conditions, aberrant lipid metabolism can lead to lipid peroxidation and trigger ferroptosis, representing a novel mechanism of oxidative-mediated cell death [8]. These findings collectively underscore the significance of redox homeostasis manipulation for cancer therapy and pave the way for the development of novel nanoplatforms with enhanced therapeutic efficacy.

**Scheme 1:**
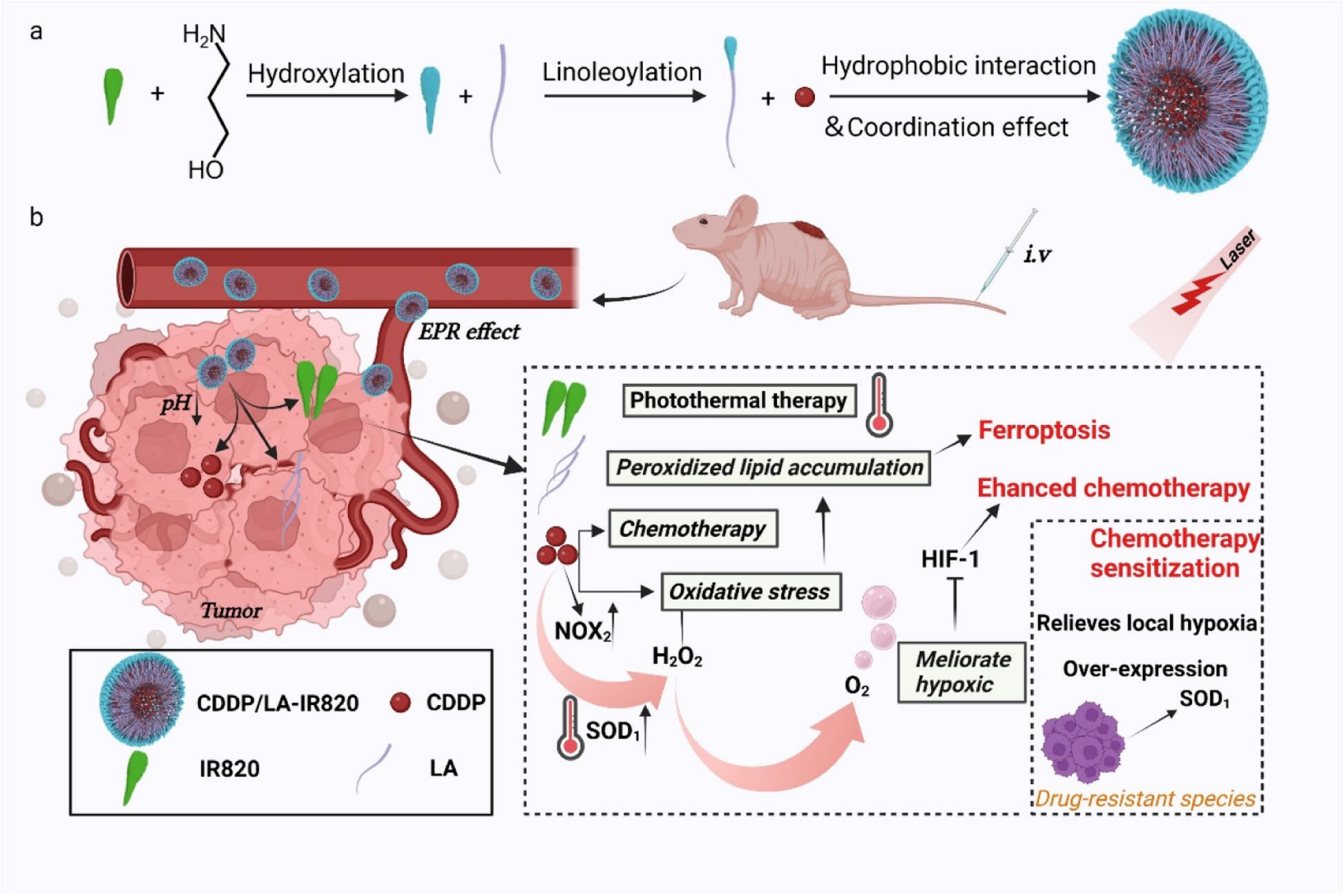
**a** Preparation of C820 NPs. **b** Schematic illustration of the underlying mechanism of C820 NPs for enhancing CDDP-IR820 photochemotherapy sensitivity

Inspired by this, we subtly designed a light-activatable oxygen self-supplying chemo-photothermal nanoplatform (C820, Scheme 1) by co-assembling first-line chemotherapeutics cisplatin (CDDP) and linoleic acid (LA)-tailored photothermal agent IR820. Light stimuli-responsive remote control is a method for manipulating specific events, processes, or functionalities within a biological system. It offers non-invasiveness, controllability, non-toxicity, and high spatiotemporal resolution [9–11]. We meticulously engineered the C820 nanoparticles as a chemo-photothermal nanoplatform, incorporating cisplatin (CDDP), linoleic acid (LA), and the photothermal agent IR820. The ester bonds were employed strategically as pivotal bridges, enabling self-supplied chemical activation and photothermal effects, and also playing a crucial role in the disassembly of the nanoparticles. Therefore, capitalizing on the enhanced permeability and retention (EPR) effect, the C820 nanoparticles efficiently accumulate at tumor sites due to their appropriate particle size. Subsequently, in the acidic tumor microenvironment, hydrolysis of the ester bond between LA and IR820-OH disrupts the hydrophobic interactions between IR820 and CDDP, leading to the disintegration of the nanoplatform. Consequently, the nanostructure exposes the therapeutic components: CDDP, photothermal agent IR820-OH, and unsaturated fatty acid LA. This sophisticated design not only facilitates the alleviation of tumor hypoxia and enhanced iron sinking but also enables a versatile synergistic effect against chemo-resistant colorectal cancer. In this nanoplatform, CDDP can produce hydrogen peroxide in CRC cells through a series of enzymatic reactions and subsequently release oxygen under laser-triggered photothermal to alleviate hypoxia. Additionally, the introduced LA can add exogenous unsaturated fatty acids into CRC cells, triggering ferroptosis via oxidative stress-related peroxidized lipid accumulation. Meanwhile, the photothermal effect can efficiently boost the rate of enzymatic response and local blood flow, hence increasing the oxygen supply and oxidizing LA for enhanced ferroptosis. As a result, this nanoplatform exhibited excellent anti-tumor efficacy in chemo-resistant cell lines and showed potent inhibitory capability in nude mice xenograft models. Taken together, this nanoplatform provides a promising paradigm via enhanced ferroptosis and alleviated hypoxia tumor microenvironment against CRC chemo-resistance.

## Results

### Preparation and characterization of C820

For the synthesis of L820, in which IR820 is connected to LA, IR820 was first hydroxylated to supply the hydroxyl group of the esterification reaction. Proton nuclear magnetic resonance (^1^ H NMR) and high-resolution mass spectrometry (HRMS) were used to analyze it (Fig. S1-B, C Supporting Information). The characteristic peak of hydroxyl (-OH) was 3.5 ppm in ^1^ H NMR and 898.37613 in HRMS, indicating that the synthesis of IR820-OH was effective. Subsequently, the hydroxylated IR820 was polymerized with LA to synthesize L820 by esterification. L820 was successfully synthesized according to the ^1^ H NMR and HRMS (Fig. S1D Supporting Information).

**Fig. 1:**
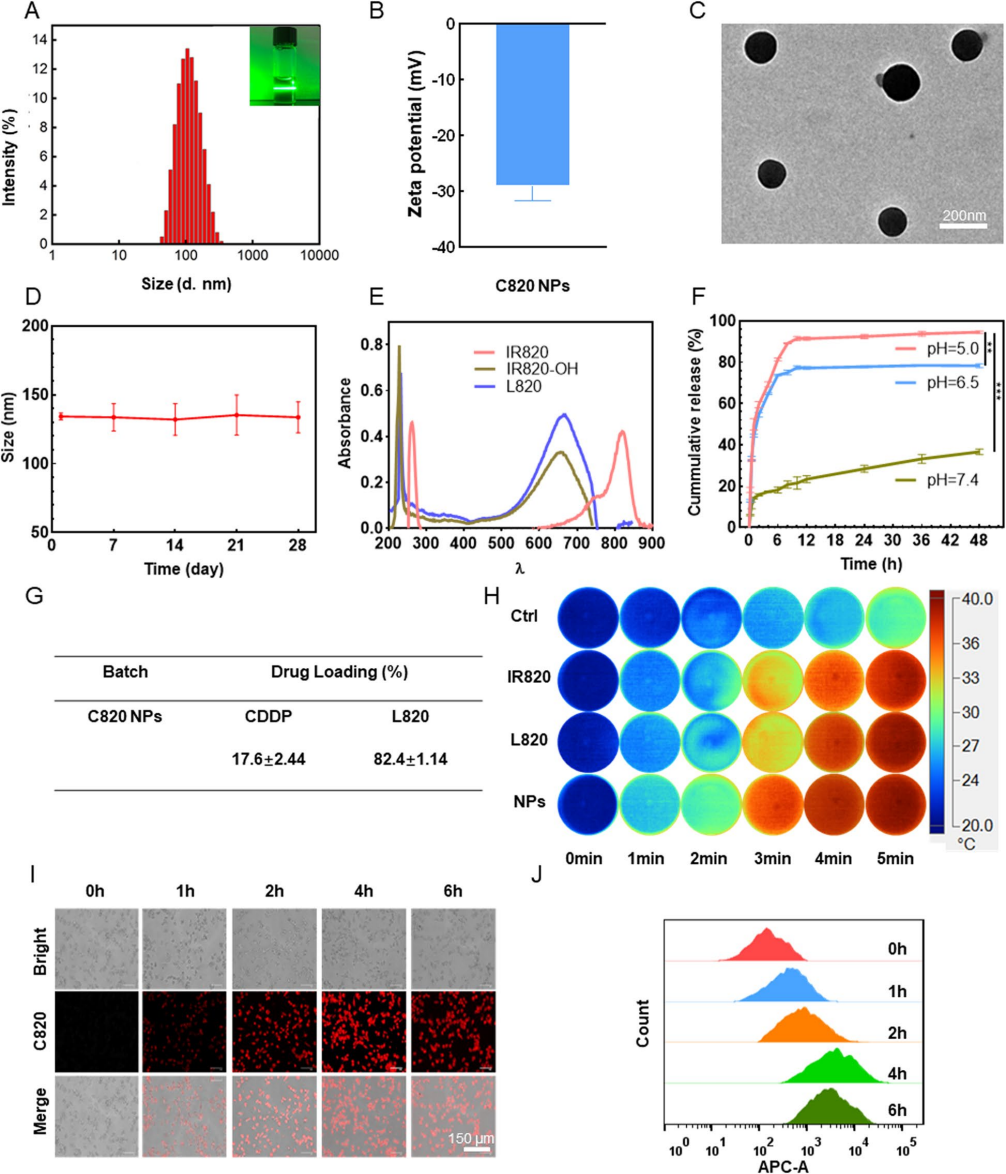
Characterization and intracellular uptake of C820 NPs. **A** Size distribution analysis and **B** zeta potentials of C820 NPs. **C** TEM image of C820 NPs. Scale bar: 200 nm. **D** Size distribution of C820/NPs over 4 weeks. **E** UV-vis absorption spectra of IR820, IR820-OH, and L820. **F** *In vitro* release profiles of CDDP from C820 NPs in PBS at pH 7.4, pH 6.5 and pH 5.0, respectively. For B, D, and F, the depicted results are mean ± SD, *n* = 3. **G** Drug loading efficiency of C820 NPs. **H** Infrared thermal images and temperature variations of IR820, L820, and C820 NPs in water after laser irradiation (Ctrl and IR820: 808 nm; L820 and C820 NPs: 660 nm; *P* = 1.0 W/cm^2^; irradiation time = 5 min). **I** Fluorescence microscopy images of C820 NPs absorbed by LoVo/CDDP at 0, 1, 2, 4, and 6 h. Scale bar: 150 μm. **J** Flow cytometry analysis of the time-dependent cellular absorption of C820. Data represent means ± SD (*n* = 3, one-way ANOVA, *P < 0.05, **P < 0.01, ***P < 0.001)

To construct NPs with stable structure, CDDP and L820 were co-assembled through hydrophobic interaction and coordination effect to form the final product C820, which was stable in an aqueous solution at a 1:1 ratio. The hydrodynamic diameter was 131.6 ± 15.3 nm (Polymer dispersity index was 0.31 ± 0.08), which had a clear Tyndall effect (Fig. 1A), and the Zeta potential was − 27.7 ± 1.5 mV (Fig. 1B). C820 was shown to be uniformly spherical in an aqueous solution using transmission electron microscopy (TEM) (Fig. 1C) [12, 13]. In physiological environment (pH = 7.0 ~ 7.4), the degradation of nanoparticles was slow, and the size of nanoparticles remained relatively stable for over 4 weeks in PBS with pH of 7.0 (Fig. 1D). UV-vis spectroscopy measurements showed that the maximum absorption wavelength of IR820-OH and L820 was 660 nm, while free IR820 was 808 nm. The blue-shift of the absorption peak further confirmed the successful synthesis (Fig. 1E). The strong absorption in the near-infrared range indicated that it had phototherapy potential, and it was more practical to employ a 660 nm laser for C820 irradiation.

To investigate the pH-responsive release capacity of C820 NPs in the acidic tumor environment, as shown in Fig. 1F and Fig. S1E, the 48-hour release rates of CDDP from C820 NPs were 35.7 ± 0.34%, 77.3 ± 0.29% and 94.2 ± 0.86% at pH 7.4, pH 6.5 and pH 5.5, respectively, demonstrating that NPs have a good pH-responsive disassembly ability. The release peak lagged behind in neutral environment, showing the ability of C820 NPs to prolong circulation time, reduce clearance rate, and control drug release at tumor sites. As shown in Fig. S1E, the release profile of IR820-OH was similar to that of CDDP, again confirming the successful synthesis and release behavior of the C820 NPs. ICP-MS (inductively coupled plasma mass spectrometry) and UV-vis were employed to measure CDDP and L820 content in these nanoparticles. The related loading efficiency were shown in Fig. 1G. The CDDP-loading efficiency of C820 NPs was calculated as 17.6 ± 2.24% and L820-loading efficiency of C820 NPs was 82.4 ± 1.14%. The degree of temperature increase following laser irradiation was tested in vitro to determine the photothermal efficacy of C820 NPs in photothermal therapy (PTT). For comparison, phosphate-buffered saline (PBS) and free IR820 and L820 were employed as control groups. PBS and IR820 were irradiated at 808 nm for 5 min, whereas L820 and C820 NPs were irradiated at 660 nm. After being exposed to near-infrared light, the temperature changes of each material group are intuitively depicted in Fig. 1H. The heating capacity is related to the concentration, indicating that the photothermal effect is concentration-dependent during the test interval (Fig. S1H, I and J). Additionally, the photothermal effects of IR820, L820, and C820 NPs under laser irradiation were comparable, suggesting that hydroxylation and LA modification have little effect on the photothermal properties of IR820.

Furthermore, excellent intracellular uptake is a crucial prerequisite for nanoparticle efficient disassembly and functioning. To evaluate cellular uptake, we utilized three colorectal cancer cell lines: CDDP-resistant LoVo (LoVo/CDDP), LoVo, and RKO. Fluorescence images showed that the cellular absorption of C820 NPs was time-dependent at the same dose and reached its maximum after four hours (Fig. 1I, S2A and C), which was consistent with flow cytometry (Fig. 1J, S2B and D). All of these results demonstrated that the developing nanoplatform C820 had high chemo-photothermal anti-tumor potential due to its good cellular uptake, pH-responsive release, and photothermal properties.

### Cytotoxicity and anti-colorectal cancer effect assessments in vitro

**Fig. 2:**
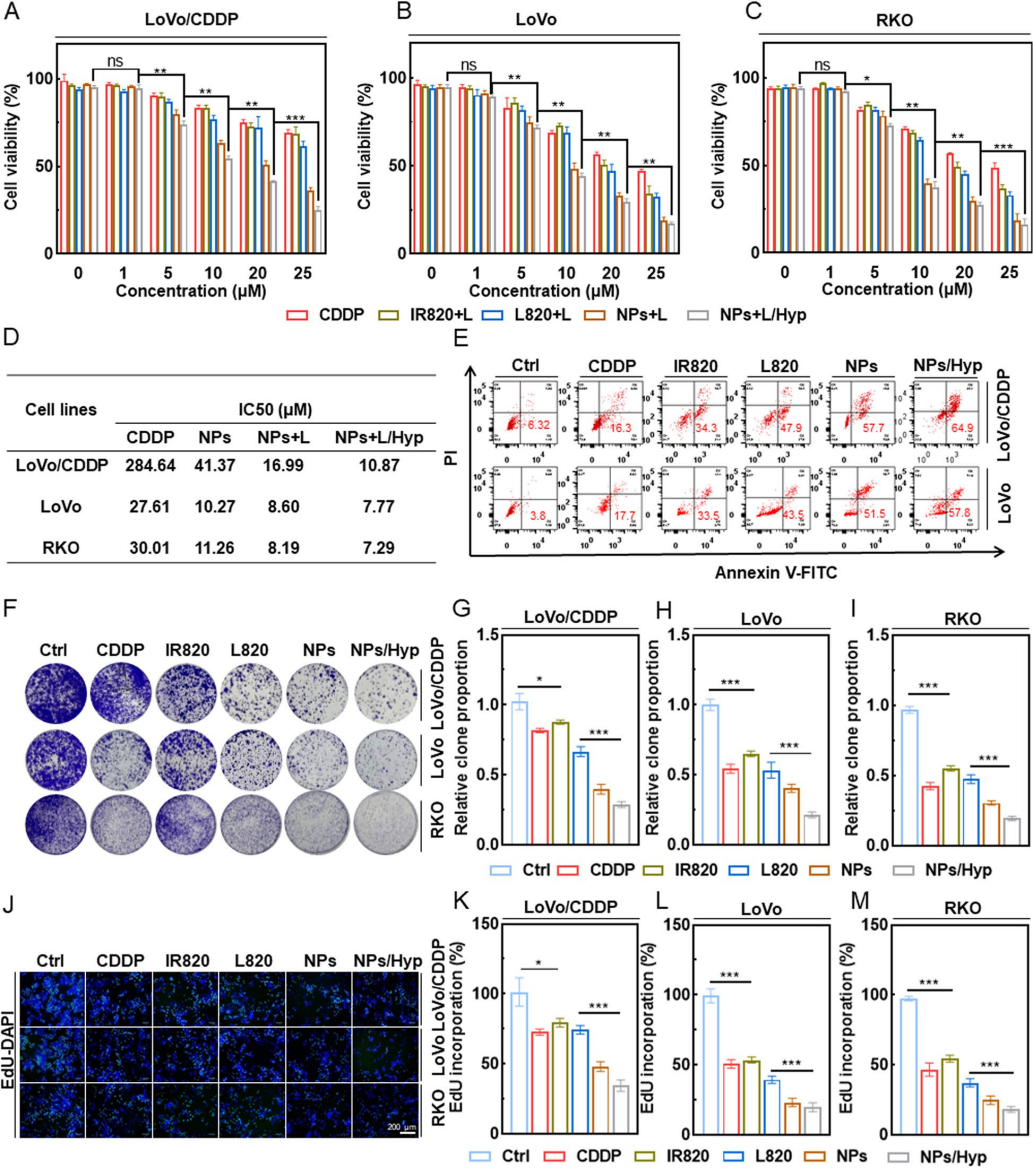
The anti-colorectal cancer effect of C820 NPs. **A**-**C** Viability of LoVo/CDDP, LoVo, and RKO cells co-cultured with CDDP, IR820, L820, C820 NPs, and C820 NPs/Hyp under laser irradiation. **D** IC50 of drugs in different groups of LoVo/CDDP, LoVo, and RKO cells with laser irradiation. **E** Apoptosis analysis in LoVo/CDDP, LoVo cells treated with PBS, CDDP, IR820, L820, C820 NPs, and C820 NPs/Hyp by Annexin V-FITC/PI double staining and quantification of the proportion of apoptotic cells. (Ctrl and IR820: 808 nm; L820 and C820 NPs: 660 nm; *P* = 1.0 W/cm^2^; irradiation time = 60 s). **F**-**I** Representative images and quantification of colony formation assay of LoVo/CDDP, LoVo, and RKO cells treated with PBS, CDDP, IR820, L820, C820 NPs, and C820 NPs/Hyp. **J** Representative images of EdU assay of LoVo/CDDP, LoVo, and RKO cells. Scale bar: 100μm. (K-M) The statistical analysis of the rate of EdU incorporation in LoVo/CDDP (**K**), LoVo (**L**), and RKO (**M**) cells). Data represent means ± SD (*n* = 3, one-way ANOVA, *P < 0.05, **P < 0.01, ***P < 0.001)

Subsequently, in order to quantify the in vitro anti-colorectal cancer efficacy of the NPs, the cytotoxicity of different formulations (CDDP, free IR820, L820, C820 NPs, and C820 NPs under hypoxia environment, abbreviated C820 NPs/Hyp) with or without laser irradiation was investigated against three different colorectal cell lines (LoVo/CDDP, LoVo and RKO) by MTT (3(4,5-dimethylthiazol-2-yl)-2,5-diphenyltetrazolium bromide). As shown in Fig. 2A-C and Supplementary S2A-C, each experimental group inhibited survival in all colorectal cancer cells in a dose-dependent manner. Notably, the cell survival rate of the C820 NPs treatment group was lower than that of any single agent, regardless of the presence or absence of light treatment, and this death effect was more significant in an environment mimicking intratumoral hypoxia. The IC50 values confirmed that the anti-tumor effect of C820 NPs was significantly superior to those of PTT and CDDP in the presence of hypoxia and laser (Fig. 2D, S2H). In contrast, free IR820, L820, and CDDP exhibited moderate cytotoxicity. Even without laser, C820 NPs/Hyp significantly decreased the IC50 values of the resistant cell lines, suggesting that C820 NPs can be effectively internalized by colorectal cancer cells and released CDDP and IR820 to achieve photo-chemotherapy and therapeutic sensitization (Fig. S2H). In addition, apoptotic cell death was assessed by flow cytometry using propidium iodide (PI) and Annexin-V fluorescein isothiocyanate (FITC) double-staining (Fig. 2E). As illustrated in Fig. 2E, the percentage of apoptotic cells was significantly higher in the C820 NPs and C820 NPs/Hyp groups than in the CDDP, IR820, and L820 groups. Nanoparticles exhibited a higher synergistic effect than monotherapies in inducing apoptosis in both cell lines in vitro. Consistent with the above results, the colony-formation assay (Fig. 2F-I) and EdU assay (Fig. 2J-M) demonstrated that C820 NPs treatment in a hypoxic environment significantly inhibited the proliferation of tumor cells. These findings indicated that C820 NPs could improve the targeted utilization efficiency of CDDP and IR820, allowing the combination of chemotherapy and photothermal therapy to achieve synergistic anti-tumor effects.

### In vitrovalidation of C820 NPs causing ferroptosis in colorectal cancer cells by inducing oxidative stress and lipid accumulation

**Fig. 3:**
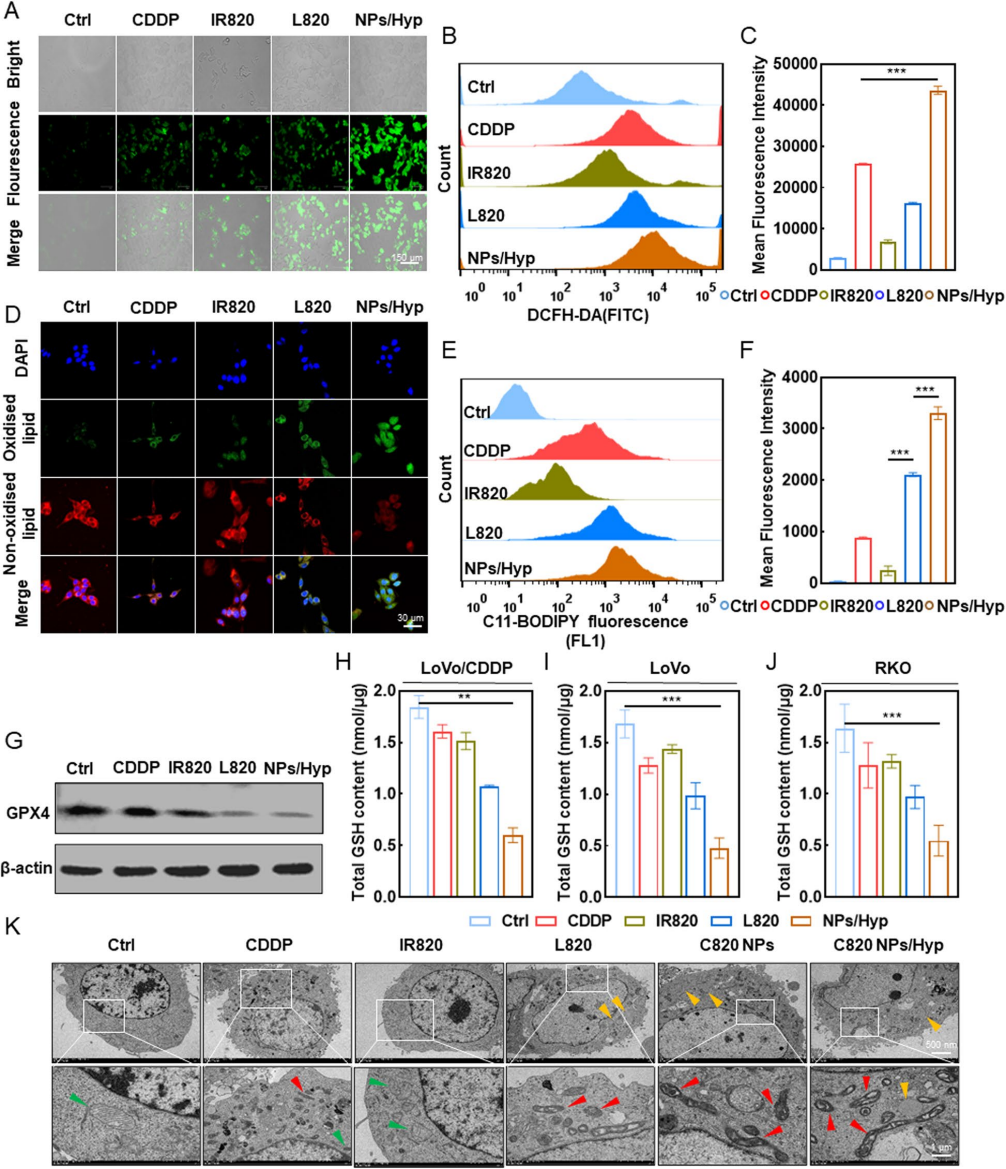
C820 NPs trigger ROS-dependent ferroptosis in colorectal cancer cells. **A** Fluorescence microscopy images and (**B** and **C**) flow cytometry quantitative analysis for intracellular ROS generation of LoVo/CDDP cells using DCFH-DA as a probe. Scale bar: 150 μm. (Ctrl and IR820: 808 nm; L820 and C820 NPs: 660 nm; *P* = 1.0 W/cm^2^; irradiation time = 60s). **D** Confocal laser scanning microscopy（CLSM）images and (**E** and **F**) flow cytometry quantitative analysis of the C11-BODIPY (581/591) probe detected lipid peroxidation. Scale bar: 20 μm. (λ = 808 nm for IR820 and 660nm for L820 and C820 NPs, *P* = 1.0 W/cm^2^; irradiation time = 60s). **G** Immunoblot analysis of ferroptosis markers (GPX4) in LoVo/CDDP cells treated as indicated. **H**-**J** GSH levels were determined after drugs and irradiation treatment. **K** Representative transmission electron microscopy images of C820 NPs-induced ferroptosis in colorectal cancer cells. Scale bar: 1 μm/500 nm. (λ = 808 nm for IR820 and 660nm for L820 and C820 NPs, *P* = 1.0 W/cm^2^; irradiation time = 60s). Data represent means ± SD (*n* = 3, one-way ANOVA, **P < 0.01, ***P < 0.001)

We explored whether the cytotoxicity of C820 NPs is related to cellular ROS overload or ferroptosis, based on the prior NPs construction premise that the means of cell death may comprise direct induction of cell death by higher ROS as well as ROS-mediated ferroptosis [14]. A hypoxic treatment was employed to mimic the hypoxic environment of the tumor. First, using the ROS-sensitive fluorescent probe 2’, 7’-dichlorofluorescein diacetate (DCFH-DA) to monitor cellular oxidative stress, we found that the fluorescence intensity was considerably higher in all cell lines treated with C820 NPs compared to monotherapy and the L820 group, under the appropriate laser irradiation even under hypoxic induction (Fig. 3A and S3A). Flow cytometry further corroborated these results (Fig. 3B, C and S3B, C). Since ferroptosis is characterized by the accumulation of lipid peroxidation products, we utilized confocal laser scanning microscopy (CLSM) to determine whether a synergistic effect exists between high ROS levels and exogenous addition of unsaturated fatty acids (LA), using C11-BODIPY (581/591) [4,4-difluro-5-(4-phenyl-1,3-butadienyl)-4-bora-3a,4a-diaza-s-indacene-3-undecanoic acid] as the lipid peroxidation sensor probe (Fig. 3D and S3D). The results suggest that the level of lipid peroxidation was remarkably evaluated following C820 NPs treatment under hypoxic conditions and maximum excitation wavelength irradiation (Fig. 3D and S3D). Flow cytometry data indicated a similar synergistic effect (Fig. 3E, F and S3E, F), as the lipid peroxidation caused by the L820 group was more than that of the monotherapy, but still less pronounced than that of C820 NPs. More importantly, the hypoxic environment did not interfere with the production of ROS or the induction of lipid peroxidation by C820 NPs.

Ferroptosis is characterized by glutathione peroxidase 4 (GPX4) deactivation and GSH levels reduction [15]. Therefore, we measured the change in GPX4 expression and GSH levels in the three cells treated with drugs and lasers or hypoxia environment. Western blots analysis showed that GPX4 protein expression levels were significantly downregulated in the C820 treatment group compared to the control group (Fig. 3G and S3G). Similarly, C820 NPs and laser therapy on total intracellular glutathione content revealed a significant decrease compared to other control groups (Fig. 3H-J). In addition, ferroptosis is manifested by organelle morphological abnormalities, especially mitochondrial modifications. Using TEM, we examined the morphological changes of cells treated with drugs and lasers [16]. Colorectal cells treated with C820 NPs exhibited ferroptotic features, including mitochondrial pyknosis, decreased mitochondrial cristae, and increased bimolecular membrane density (Fig. 3K and S3H). In contrast, the CDDP and L820 groups displayed cell structure alterations, whilst the free IR820 group showed a much weaker change. Moreover, significant intracellular lipid droplets were observed in the drug-treated group following LA introduction. Overall, these results indicate that CDDP and LA-modified IR820 have strong synergistic effects in inducing ferroptosis and killing tumor cells.

**Fig. 4:**
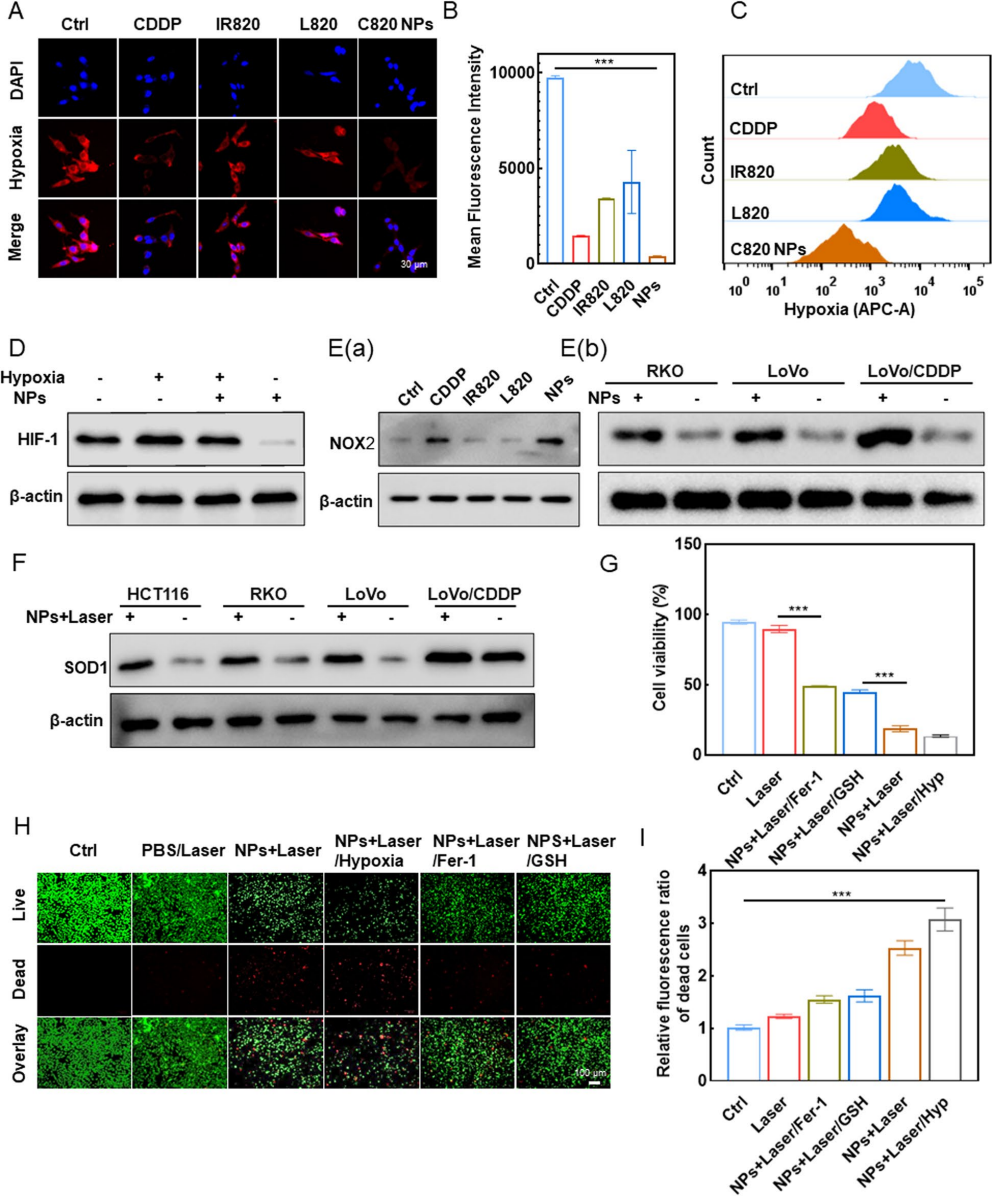
Enhancing photochemotherapy synergistic sensitivity through improving tumor cell hypoxia. **A** CLSM images of LoVo/CDDP cells treated accordingly and stained with hypoxia probes in the presence of 1% O_2_. Scale bar: 30 μm. **B** Statistical analysis of mean fluorescence intensity in LoVo/CDDP cells treated with hypoxia and different drugs. **C** Flow cytometry analysis of intracellular hypoxia level in LoVo/CDDP cells after different treatments in a hypoxic environment. **D** Immunoblot analysis of hypoxia markers in LoVo/CDDP cells treated with PBS or C820 NPs with or without hypoxia induction. **E** Immunoblot analysis of NOX2 in LoVo/CDDP cells treated with PBS, CDDP, free IR820, L820, and c820 (Ea) or in RKO, LoVo, and LoVo/CDDP cells treated with C820 NPs (Eb). **F** Immunoblot analysis of SOD1 in RKO, LoVo, and LoVo/CDDP cells treated with or without C820 NPs and laser irradiation. **G** Effects of NPs, laser, hypoxia and ferroptosis inhibitors on LoVo/CDDP cells viability. **H** Fluorescence microscopy images showing LoVo/CDDP cell survival after different treatments. Live cells are marked with calcein-AM (green fluorescence), while dead cells are marked with propidium iodide (red fluorescence). Scale bar: 100 μm. **I** Statistical analysis of the relative fluorescence ratio of dead cells treated as indicated. Data represent means ± SD (*n *= 3, one-way ANOVA, **P < 0.01, ***P < 0.001)

Hypoxia is a fundamental feature of the vast majority of solid tumors and can speed up malignant progression, tumor cell proliferation, and metastasis [17]. Here, we study how C820 NPs improve tumor cell hypoxia. Preliminary evidence suggests the CDDP- and photothermal agent IR820-containing C820 nanoparticle can ameliorate tumor hypoxia. We used CLSM to demonstrate that C820 NPs may create O_2_ in response to NIR irradiation to reverse hypoxia and improve the efficacy of photochemotherapy in hypoxic LoVo/CDDP and LoVo parental cells. Intracellular hypoxia was detected using a kit that converts the nitro group of the hypoxia probe to hydroxylamine and amino groups in the hypoxic intracellular environment, resulting in faint to brilliant red fluorescence. As shown in Fig. 4A, under hypoxic conditions (1% O_2_), control group cells showed a prominent red fluorescence, which was significantly diminished in the C820 NPs treatment group, indicating that C820 NPs could indeed produce oxygen under laser irradiation. Other single-agent treatment groups similarly improved hypoxia intensity, but the impact was less satisfactory, revealing the superior synergistic activity of C820 NPs. Flow cytometry analysis of the fluorescence intensity of the hypoxia probe supported these findings (Fig. 4B and C).

Previous studies indicate that tumor cells adapt to hypoxia by activating the hypoxia-inducible factor 1 (HIF-1), which drives the expression of numerous growth factors and cytokines [18, 19]. To investigate the pharmacological effects of C820 NPs in CRC, we analyzed the global expression changes systematically and identified that C820 NPs and laser treatment significantly downregulated HIF-1 (Fig. 4D). CDDP can specifically activate nicotinamide adenine dinucleotide phosphate oxidase (NADPH oxidase, NOX) in cancer cells. NOX catalyzes the oxidation of NADPH to NADP^+^ and releases an electron to an O_2_ molecule, yielding O_2_^•−^, which is then dismutated into H_2_O_2_ and O_2_ by superoxide dismutase [20, 21]. Therefore, to confirm that CDDP and C820 NPs indeed have the ability to specifically up-regulate NOX protein expression, we next investigated the fluctuation in the expression level of a representative protein of the NOX family, NOX2, after receiving different drugs, verifying the ability of C820 NPs to specifically up-regulate the expression of NOX2 protein in multiple cell lines. As shown in Fig. 4E(a-b), the expression level of NOX2 was significantly up-regulated in CDDP or C820 NPs treated groups compared to other control groups, and the results were consistent with those reported in the literature, indicating the ability of C820 NPs to enhance NOX2 expression specifically. SOD, another crucial enzyme involved in CDDP-induced oxygen synthesis, is also an important component of the anti-redox system, and its expression level fluctuates in cells when exposed to high temperatures or oxidative stress [22, 23]. There are three isozymes of SOD in mammals, including the cytoplasmic Cu/Zn-SOD (SOD1), the mitochondrial SOD (SOD2), and the extracellular SOD (ECSOD or SOD3). In this study, we indirectly showed the intensity of the oxygen production reaction in the cytosol by measuring the expression level of SOD1. After treatment with C820 NPs and laser irradiation, SOD1 protein expression levels were significantly up-regulated in a variety of CRC cells, indicating cellular antioxidant defense (Fig. 4F). Notably, the resistant LoVo cell line (LoVo/CDDP) had much greater baseline expression levels of SOD1, which could be one of the reasons of its resistance. These findings support that C820 NPs improve photochemotherapy efficacy and overcome drug resistance by ameliorating environmental hypoxia.

To further elucidate the mechanism that C820 NPs kill tumors by triggering hypoxia reversal and oxidative stress-related cell ferroptosis, we compared cell survival in hypoxia and normoxia, as well as the effect of iron death pathway inhibitors. MTT assay results showed that C820 NPs had a higher inhibitory impact in an anoxic environment than in cells cultured in a normal oxygen environment under the same conditions (Fig. 4G). Interestingly, ferrostatin-1 (Fer-1) and GSH dramatically suppressed C820 NPs and laser-induced cell death (Fig. 4G). The results were further confirmed by live/dead cell staining, where live cells appeared green and dead cells showed red under fluorescence microscopy (Fig. 4H and I). In summary, C820 NPs with both chemotherapeutic and photothermal effects can significantly induce ferroptosis in colorectal cancer cells and specifically stimulate the up-regulation of CDDP-related oxygen production key enzymes, which can improve the anti-tumor efficiency by improving hypoxia, which may be more obvious in cells resistant to oxidative stress. C820 NPs combined with NIR treatment induced ferroptosis and enhanced CRC cell sensitivity to photochemotherapy by improving hypoxia.

### In vivo biodistribution and anti-tumor ability of C820 NPs

**Fig. 5:**
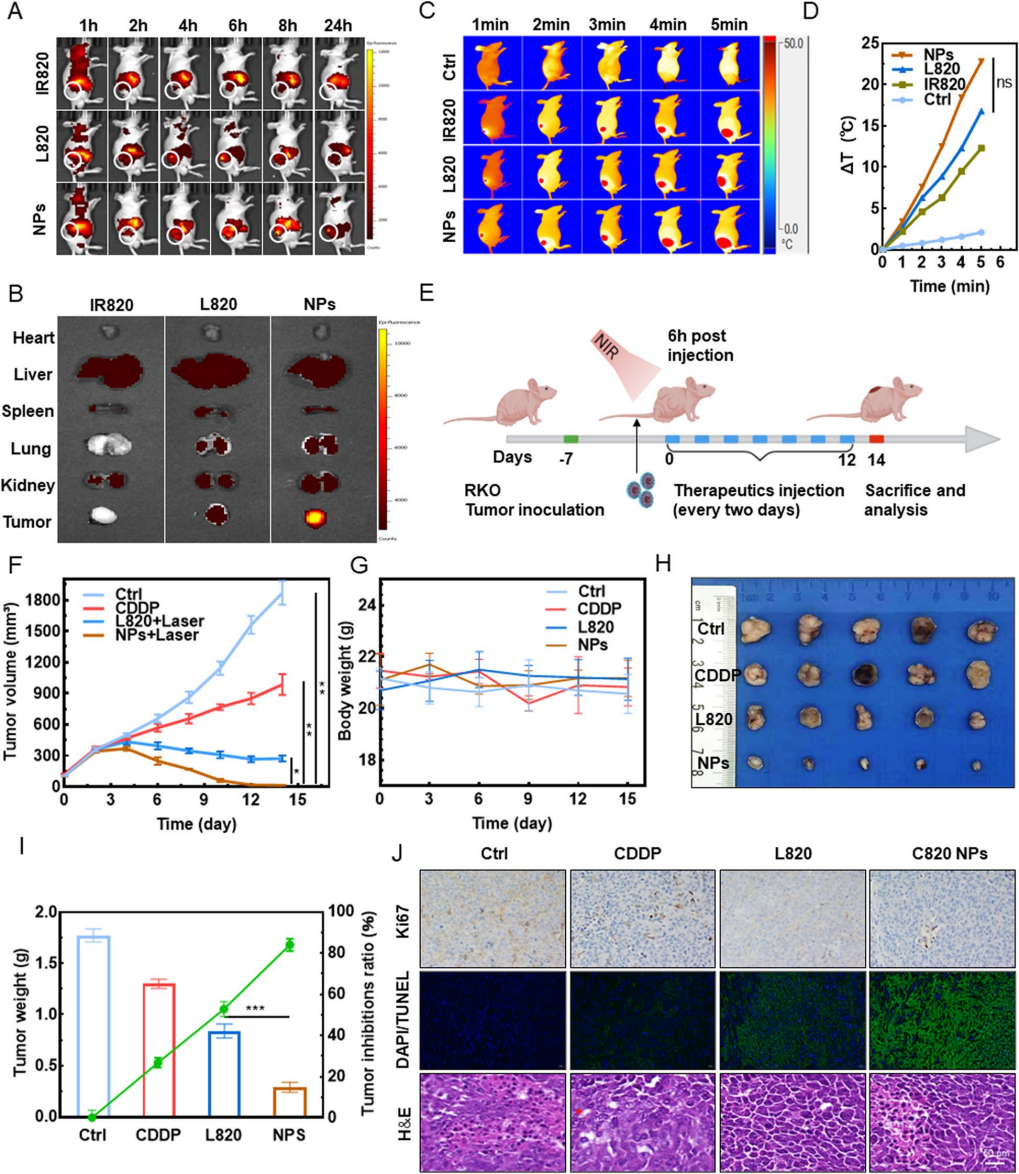
*In vivo* anti-tumor efficacy evaluation. **A** Fluorescence imaging and whole-body biodistribution of IR820, L820, and C820 NPS at different time points in the RKO tumor-bearing BALB/c nude mice model. **B** Fluorescence signal images of main excised organs (heart, liver, spleen, lung, kidney, and tumor) in mice treated with IR820, L820, and C820 NPs at 24h post injection. **C** and **D** Infrared thermal images and corresponding temperature variation graph of RKO tumor-bearing nude mice subjected to different treatments followed by laser irradiation for various durations (1, 2, 3, 4, and 5 min; Ctrl and IR820: 808 nm; L820 and C820 NPs: 660 nm; *P* = 1.0 W/cm^2^. **E** Schematic illustration of *in vivo* treatment on tumor-bearing nude mice. **F** Tumor volume change graph. **G** Weight change line chart. **H** Representative image of isolated tumor from nude mice. Scale bar: 1 cm. **I** Final tumor volumes and the inhibition rate. **J** Ki67, TUNEL, and H&E staining of the tumors at the end of anti-tumor studies. Scale bar: 150 μm. All data are shown as mean ± SD and are representative of five independent experiments; *n*=5, one-way ANOVA, ^ns^ *P*＞0.05, *P < 0.05, **P < 0.01, ***P < 0.001

Using a near-infrared fluorescence (NIRF) imaging system, C820 NPs were used as fluorescent probes to track their biodistribution and tumor accumulation in vivo in real time to ensure adequate therapeutic performance (Fig. 5A). The statistics of fluorescence (Fig. S4A) show that both free IR820, L820, and C820 NPs reached maximum fluorescence after 6 h, hence laser irradiation at 6 h following intravenous administration was chosen for subsequent PTT. Moreover, C820 NPs fluorescence signal lasted 24 h longer than free IR820 and L820 at tumor sites. The tumor and main organs were excised to determine accumulation efficiency after 24 h (Fig. 5B). C820 NPs retained longer in the tumor site than free IR820 or L820, indicating that the enhanced permeability and retention (EPR) effect may promote the accumulation of C820 NPs at the tumor site (Fig. S4B). To investigate the photothermal effect of C820 NPs in vivo, infrared thermal imaging of the tumor was used (Fig. 5C). The established tumor-bearing mice were injected through the tail vein with normal saline (NS), CDDP, IR820, L820, and C820 NPs. All IR820-contained groups showed a significant photothermal effect at 6 h after administration, especially the C820 NPs group elevate the temperature by approximately 32 ℃ in 5 min of exposure, while the NS group increasing by less than 3 ℃, demonstrating IR820-contained drugs capacity to thermally ablate cancer cells when exposed to NIR laser (Fig. 5C and D). The heating amplitude of the L820 group was slightly higher than that of the IR820 group, implying that LA delayed IR820’s departure from the body. The C820 NPs group had a greater temperature than the IR820 and L820 groups, possibly due to the stronger tumor-targeted enrichment of the NPs.

Next, we performed in vivo tumor inhibition experiments using mice bearing RKO cell xenografts. Since C820 NPs or L820 are biologically capable of outperforming IR820 and being more effective, mice with RKO cell xenografts were injected with saline, CDDP, L820, and C820 NPs via the tail vein every 2 days for 12 days to observe the in vivo therapeutic effect (Fig. 5E). Changes in tumor growth during the therapy period were shown in Fig. 5F. After 15 days, there were no noticeable changes in mouse body weight, suggesting no obvious biotoxicity for the evaluated drugs (Fig. 5G). Finally, mice were euthanized, and tumors were extracted and weighed (Fig. 5H and I). The tumor inhibition ratios for NS, CDDP, IR820, L820, and C820 NPs were close to zero, 26.8%, 52.8%, and 84.0%, respectively (Fig. 5I). Among all groups, the C820 NPs group had the smallest tumor volume and the highest tumor inhibition ratio. According to Ki-67 analysis, C820 NPs showed a good anti-proliferative effect on the tumor, and laser irradiation significantly enhanced the inhibition of proliferation by activating PTT and ferroptosis (Fig. 5J). Terminal deoxynucleotidyl transferase-mediated dUTP-biotin nick end labeling assay (TUNEL) and Hematoxylin-Eosin (H&E) staining of tumor tissues also supported C820 NPs’outstanding anti-tumor effects (Fig. 5J). Blood biochemical indexes (Figure S4A-E, Supporting Information) and H&E staining of the main organs (Figure S4F, Supporting Information) indicated undetectable toxicity to mice at the tested dose, demonstrating the great therapeutic biosafety of C820 NPs in cancer treatment. Taken together, these results imply that C820 NPs exert promising tumor suppressive effects both in vitro and in vivo.

### C820 NPs enhanced the therapeutic sensitivity of cisplatin-resistant tumors and inhibited tumor recurrence in vivo

**Fig. 6:**
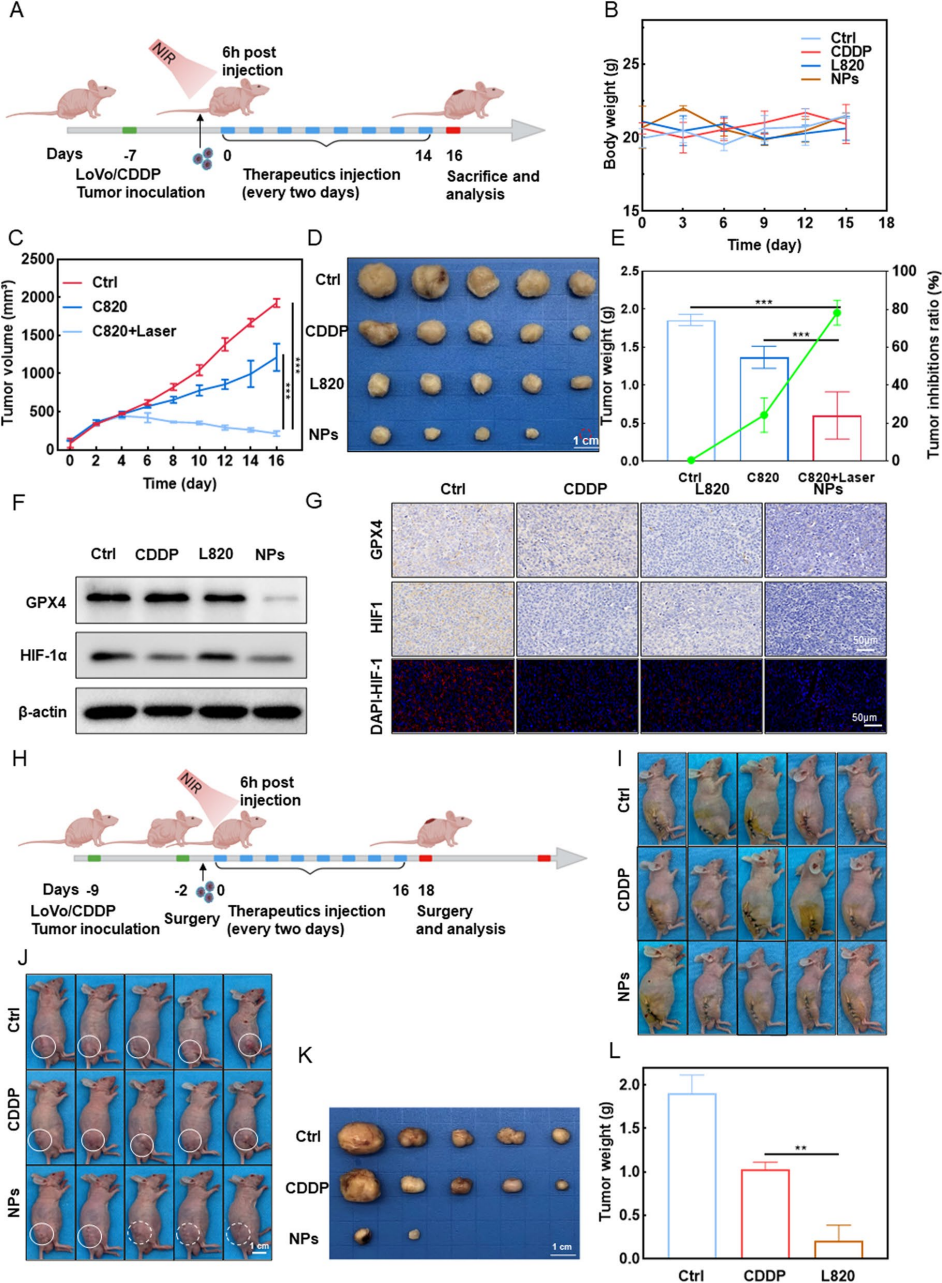
C820 NPs sensitized cisplatin chemotherapy and inhibited recurrence. **A** Schematic illustration of the establishment and treatment regimen for the LoVo/CDDP tumor-bearing BALB/c nude mice model. **B** Body weight of tumor-bearing BALB/c nude mice (*n* = 5) in different groups. **C** Tumor volume and **D** representative images of the dissected tumors of different groups (*n* = 5). **E** Tumor weight and inhibition ratio of tumor-bearing BALB/c nude mice (*n* = 5) with different treatments. **F** The expression levels of GPX4 and HIF-1 protein in tumors by western blot. **G** Representative immunohistochemistry and immunofluorescence images of tumor sections from control and treated mice. **H** Schematic illustration of tumor recurrence model establishment and subsequent treatment. **I** and **J** Representative images of mice that relapse on the day and after surgery (*n* = 5). **K** Tumor weight and inhibition ratio of recurrent tumors. **L** Final tumor weights (*n* = 5). All data are shown as mean ± SD and are representative of five independent experiments; one-way ANOVA, *P < 0.05, **P < 0.01, ***P < 0.001

We predict that C820 NPs have the same significant anti-tumor effect in drug-resistant tumor-bearing mice based on their excellent targeting, photothermal and anti-tumor effects in vivo. Concerning CDDP-resistant tumors that differentially express key genes in the CDDP-generated oxygen cascade, a LoVo/CDDP tumor-bearing BALB/c nude mouse model was established to assess the ability of C820 NPs to overcome drug resistance in vivo (Fig. 6A). Near-infrared fluorescence (NIRF) and infrared thermal imaging revealed that the biodistribution and photothermal capacity of C820 NPs in vivo were similar to those of RKO tumor-bearing mice (Fig. S5A-D). Seven days after inoculation, 2 mg/kg of saline, free CDDP, L820, and C820 NPs were administered intravenously into the tail vein of the mice (*n* = 5 for each group) every 2 days for 14 days. The tumor size and body weight were measured every three days during treatment. As a result, mice in each treatment group experienced minor changes in body weight throughout the study, indicating the good biosafety of the nano-strategy (Fig. 6B). As expected, CDDP-resistant tumor-bearing mice treated with C820 NPs exhibited tumor suppression similar to that of sensitive cells, with the highest tumor suppression rate, while the free CDDP group showed treatment resistance (Fig. 6C-E). Total tumor tissue protein was then extracted for western blot analysis of GPX4 and HIF-1 expression levels (Fig. 6F). C820 NPs decreased the expression of GPX4 and HIF-1 protein in the tissue, corresponding with the in vitro findings. To further elucidate the mechanism by which C820 NPs enhance CDDP sensitivity in vivo, immunohistochemistry and immunofluorescence staining of GPX4 and HIF-1 expression in LoVo/CDDP tumor tissues was performed (Fig. 6G). GPX4 and HIF-1 expression was dramatically decreased in the C820 NPs group, suggesting specific ferroptosis and amelioration of the hypoxic environment in tumor tissues following C820 plus laser treatment, hence promoting tumor regression. Moreover, histopathological examination of the tumor and vital organs (heart, liver, spleen, lungs, and kidney) with H&E staining revealed that the anti-tumor activity of C820 NPs + laser treatment was optimal and did not show any apparent pathological damage or lesion, and serum biochemical indicator (CK-MB for the heart, ALT/AST for the liver, and BUN/CRE for the kidney) assays showed normal fluctuations in these groups, suggesting that C820 NPs are biocompatible without causing any evident toxicity to the major organs (Fig. S5E-I). Overall, the aforementioned findings demonstrated that C820 NPs have tremendous potential for use in PTT, since the ferroptosis and O_2_ induced by C820 could evidently improve the anti-tumor effect and overcome treatment resistance.

As tumor resistance is one of the leading causes of relapse in various types of cancer, there is an urgent need to understand the ability of C820 NPs to inhibit tumor recurrence [24, 25]. We used animals to imitate the clinical tumor treatment procedure to observe the effect of C820 NPs on preventing postsurgical recurrence (Fig. 6H). C820 NPs therapy significantly prevented tumor recurrence compared to the control or free CDDP, indicating that C820 NPs have the same killing potential against local or systemic tumor cells and could effectively inhibit tumor recurrence following surgery (Fig. 6I-L). We have also conducted additional experiments comparing the degree of tumor growth inhibition, as well as NOX and SOD levels in tumor tissue in the presence and absence of laser irradiation. The tumor photos, tumor volumes, and weights shown in Figure S6A-C supported that C820 inhibited the tumor growth process, with C820 + Laser treatment being the most effective. Notably, the related tumor inhibition ratio was about 80% in the C820 + Laser. The NOXs and SOD levels in the tumor tissues were also significantly increased in C820 + Laser groups compared to C820 groups (Figure S6D). Taken together, these results suggest that the C820 NPs manufactured in this study offered excellent potential for tumor therapy and overcoming drug resistance, making them a promising candidate for multifunctional drug delivery and cancer therapy.

## Discussion

Colorectal cancer (CRC) is the second most deadly cancer in the world, and its burden is projected to rise further in the coming decades [26]. The clinical management of CRC is hampered by several challenges, such as chemoresistance, hypoxia, tumor recurrence and metastasis, and intratumoral heterogeneity [26, 27].

Drug resistance is one of the main reasons for the failure of colorectal cancer treatment and postoperative recurrence [28]. Due to the inhibition of apoptosis pathway or the activation of anti-apoptosis pathway, tumor cells often escape apoptosis, thus increasing survival and tolerance [29, 30]. In addition, due to the Warburg effect, the local hypoxia of tumor can activate hypoxia-inducible factor (HIF) signaling pathway, induce angiogenesis and apoptosis inhibitors, and promote tumor progression and treatment resistance [31]. Ferroptosis is a novel mode of programmed cell death that can circumvent the apoptotic resistance of CRC cells by depleting intracellular glutathione (GSH) and down-regulating glutathione peroxidase 4 (GPX4) among other mechanisms. Moreover, the induction of ferroptosis can disrupt the metabolic balance and homeostasis of colorectal cancer cells by elevating ROS levels and lipid peroxidation, and impair their tolerance to exogenous stimuli such as chemotherapy, radiotherapy and targeted therapy [32–34]. In this study, we harness the unique thermal effects of light stimuli, effectively enhancing the biological functionality of nanomaterials and maximizing the synergistic effects of this nanoplatform [35, 36]. In addition to light stimuli, other approaches include ultrasound stimulation [37] and magnetic control [38–43]. Therefore, improving tumor hypoxia and inducing ferroptosis of tumor cells may be a feasible treatment strategy for overcoming apoptosis escape and treatment resistance in cancer therapy.

In this study, we rationally designed a light-activatable oxygen self-supplying chemo-photothermal nanoplatform by co-assembling CDDP and LA-tailored IR820 via enhanced ferroptosis against colorectal cancer chemo-resistance. In this nanoplatform, CDDP can generate H_2_O_2_ in CRC cells through a series of enzymatic reactions, which then releases oxygen under NIR triggered photothermal to alleviate hypoxia. Moreover, the introduced LA can supply exogenous unsaturated fatty acids and targeting ability to CRC cells, inducing ferroptosis by oxidative stress-related lipid peroxidation accumulation. Meanwhile, the photothermal can effectively increase the reaction rate of enzymes and local blood flow, thereby enhancing oxygen supply and oxidation of LA, and boosting ferroptosis. We demonstrated that the designed nanoplatform exerted remarkable anti-colorectal cancer effects both in vitro and in vivo, and efficiently circumvented resistance and postoperative recurrence. The underlying mechanism of its efficacy was that C820 NPs potently triggered ferroptosis of colorectal cancer cells, and reversed their apoptotic and chemotherapeutic resistance by suppressing HIF-1 expression via ameliorating tumor hypoxia. This offers a safe and effective strategy for the treatment of colorectal cancer.

To emphasize the originality of our proposed nanoparticle assembly system, we have compared our work with other studies in the field, including recent papers reported by the corresponding authors of this manuscript. Previous studies related to nanoparticle assembly, such as those by Yuri Kim et al. [44], Gunhyu Bae et al. [45], and Hyunsik Hong et al. [46], have demonstrated the versatility of this approach in constructing functional nanoplatforms. While these works explored different materials and assembly methods, our study distinguishes itself by focusing on the specific application of ferroptosis treatment in chemo-resistant CRC. A recent study by Tongyu Li et al. also employed nanoparticle assembly to develop a nanoplatform for cancer therapy [47]. However, unlike their approach, which targeted a different type of cancer, our work addresses the pressing issue of chemo-resistance in CRC. Furthermore, our study stands out due to the unique chemical modification approach used to create C820 NPs. Differing from our previous studies [48–50], our current study aims to modulate the antioxidant-reduction homeostasis for the treatment of colorectal cancer. We successfully bridged the water-soluble IR820 with the insoluble CDDP by introducing an exogenous unsaturated fatty acid (LA), resulting in enhanced bioavailability and photothermal therapy efficacy. This innovative approach allowed us to achieve robust anti-tumor effects, even in chemotherapy-resistant CRC models with high SOD1 expression. Overall, our study showcases the originality of utilizing nanoparticle assembly for developing a nanoplatform tailored for ferroptosis treatment against chemo-resistant CRC. The unique features of our assembly system and its application in combating drug resistance highlight the potential significance of this approach in cancer therapy.

Nanomedicine plays an important role in the diagnosis and treatment of cancer, using the targeting and slow-release properties of nanoparticles to improve the efficacy and safety of drugs [51], and reduce side effects [52] and toxicity [53]. In this study, the novel tumor treatment strategies realized by nanoplatforms seem to be a very promising way. However, continuous improvement and more research are needed to prove its potential for clinical promotion.

## Conclusions

In this study, we performed chemical modification for the first time of bridging the water-soluble IR820 with the insoluble CDDP into a nanoscale particle by introducing an exogenous unsaturated fatty acid (LA), which improved single-drug bioavailability and photothermal therapy efficacy. IR820 and CDDP can passively target tumor tissue through EPR effects by forming nanoparticles, potentially reducing toxicity to normal cells. Furthermore, the introduction of LA expands the synergistic tumor inhibitory effect of CDDP and IR820. In addition to exerting chemotherapy efficacy, CDDP promotes oxidative stress in tumor cells, which peroxidizes LA and other lipids and triggers ferroptosis. On the other hand, photothermal therapy boosted the cascade reaction following CDDP internalization, and the generated oxygen improved the hypoxic state in the tumor, effectively inhibiting tumor progression. This phenomenon was particularly prominent in drug-resistant tumors with high SOD1 expression. As a result, C820 NPs presented negligible side effects and robust anti-tumor effects in both chemotherapy-resistant and non-resistant CRC models. Therefore, our designed NIR light-activated oxygen self-supplying nanoplatform provides a promising strategy for combating cancer drug resistance by enhancing ferroptosis.

## Materials and methods

### Materials

Cisplatin (C295225), Linoleic acid (L100441), 3-(Methylamino)-1-propanol (M133702), triethylamine (T431604) and N, N-dimethylformamide (DMF, D112004) were purchased from Aladdin. N-hydroxysulfosuccinimide (NHS), 1-Ethyl-3-(3-dimethylaminopropyl) carbodiimide hydrochloride (EDCI) and 4-dimethylaminopryidine (DMAP) were purchased from Aladdin Reagent Co. IR820 (448,014) was obtained from J&K Scientific. 3-(4,5-dimethylthiazol-2-yl)-2,5-diphe-nyltetrazolium bromide (MTT), crystal violet (C0775), were purchased from Millipore Sigma. DAPI was purchased from Invitrogen. Ethanol and Sodium chloride injection were purchased from Sichuan Kelun Pharmaceutical Co., Ltd. The ROS assay kit (Catalog no. S0033s), Live/Dead detection kit (Catalog no. C2015M), TUNEL apoptosis assay kit (Catalog no. C1086), Lipid Peroxidation MDA Assay Kit (Catalog no. S0131S), Total Glutathione Assay Kit (Catalog no. S0052) were provided by Beyotime Biotechnology. The Hypoxia detection kit (Enzo Life Sciences Inc., Farmingdale, USA). Anti-GPX4 antibody (ab219592), anti-HIF-1 (ab51608), and anti-NOX2 antibody (ab129068) were obtained from Abcam (Cambridge, UK). Anti-SOD1 (SC11407) antibody and Anti-beta actin antibody (sc-47,778) were obtained from Santa Cruz Biotechnology.

### The preparation of C820 NPs

Synthesis of IR820-LA (L820): First, IR820 was hydroxylated. IR820, 3-amino-1-propanol, and triethylamine (dissolved in anhydrous DMF, molar ratio 1:2:4) were added sequentially to a 50 mL round bottom flask and stirred for 4 h at 85 °C with nitrogen protection and aluminum foil shade (500 rpm). After the reaction was completed, the solvent (anhydrous DMF) was removed by rotary evaporation to obtain IR820 hydroxylated crude product (IR820-OH), which was then purified by silica gel column chromatography [54]. The resultant IR820-OH is scraped and weighed to calculate the molar mass. Next, LA, NHS, EDCI, and DMAP (dissolved in anhydrous DMF, molar ratio 1:2:2:2) were sequentially added to a 50 mL round bottom flask and stirred for 30 min at 0 °C with nitrogen protection and aluminum foil shade (500 rpm). The purified IR820-OH (1 unit molar mass) was dissolved in anhydrous DMF by ultrasound, added to the above reaction solution, and stirred for 24 h at room temperature with nitrogen protection and aluminum foil shade (500 rpm). The solvent (anhydrous DMF) was removed by rotary evaporation after the reaction to produce the crude product of IR820 linoleic acidification (L820). Finally, L820 was purified by silica gel column chromatography [55].

Synthesis of C820 NPs: C820 NPs were carried out using a combination of thin film dispersion and ultrasonic dispersion [56]. Briefly, L820 and cisplatin were dissolved in a methanol solution containing 10% DMF (DMF/Methanol, 10% v/v) and fully dissolved by ultrasound. Remove the solvent by rotary evaporation, and the mixture was sonicated and dispersed into 5ml deionized water.

### The characteristics of C820 NPs

Characterization of C820 NPs: The size distribution and zeta potential of C820 were measured using a Zeta sizer Nano Analyzer (Malvern, UK). The morphology of C820 NPs was observed by a transmission electron microscope (TEM, HT7800) in Electron Microscope Room, West China School of Basic Medical Sciences & Forensic Medicine, Sichuan University. The UV–vis absorption spectra of nanoparticles were recorded at room temperature using a UV–vis spectrophotometer (UV2700, Shimadzu, Japan).

### In vitro photothermal performance

To determine the photothermal performance of the C820 NPs, 1mL of C820 NPs solution was irradiated by 660 nm laser (1.0 W/cm^2^). The temperature change of solution in 5 min (30s interval) was detected by a digital thermometer respectively and infrared thermal imager.

### The pH-responsive release capacity of C820 NPs

The release of CDDP and IR820-OH from C820 NPs was carried out by dialysis method. C820 NPs solution was placed into various dialysis bags (MWCO = 3500 Da), and each dialysis bag contained 1 mL solution. After sealed, all dialysis bags were immerged in 25 mL phosphate buffer solution (PBS; pH 7.4, 6.5 or 5.0) containing 5% (v/v) tween-80 at 37 °C under shaking at 100 rpm. Then, 1 mL release medium was withdrawn and replaced with 1 mL fresh release medium at predetermined time. The release content of IR820-OH in medium was examined by UV–vis spectrophotometer (UV-8000 S) at 660 nm, and the IR820-OH concentration was computed according to the standard curve. The release content of CDDP in medium was examined by inductively coupled plasma mass spectrometry (ICP-MS, Perkin Elmer Elan 6100). All groups were conducted in parallel triplicate.

### Cell culture

Human colorectal cancer cells LoVo, LoVo/CDDP and RKO cells were selected as tumor cell models. LoVo and LoVo/CDDP were maintained in RPMI 1640, while RKO was maintained in DMEM, supplemented with 10% FBS, 100 U/mL penicillin, and 100 U/mL streptomycin and kept in either a normoxic incubator containing 5% CO_2_ at 37℃ or a hypoxia incubator with 1% O_2_, 92% N_2_ and 5% CO_2_ at 37℃.

### Cellular uptake assay

The in vitro cellular uptake of free IR820, L820, and C820 NPs was determined by fluorescence imaging (Nikon, Tokyo, Japan) before and after NIR irradiation. The cellular uptake of C820 NPs at different time points was evaluated using the fluorescence imaging system.

### Antiproliferation assay

The conventional MTT assay was used to determine the cell viability. Briefly, 5000 cells (LoVo, LoVo/CDDP, and RKO) were seeded onto each well of a 96-well plate and incubated for 24 h at 37 °C in a normoxic or hypoxic incubator. After discarding the supernatant, the cells were washed with PBS. For the light-treated cells, the IR820 group was irradiated with an 880 nm laser for 2 min (1.0 W/cm^2^), while the L820 and C820 groups were irradiated with a 660 nm laser, and the cells were cultured for an additional 24 h. The MTT reagent (0.5 mg/mL) was added to each well and incubated for an additional 3.5 h. Following incubation, the MTT medium was withdrawn and 150 ul of DMSO was added to each well to dissolve the MTT formazan crystals. An ELISA multi-well spectrophotometer was used to detect absorbance at 490 nm test wavelength and 570 nm reference wavelength.

The long-term effects on tumor cell proliferation were analyzed using a colony formation assay, as described previously [15]. Cells were seeded in 24-well plates (500 cells/well) and treated with the indicated concentration of reagents. The medium was changed every three days. After two weeks, cell colonies were washed three times with PBS for 3 min each, fixed with 4% paraformaldehyde for 30 min, then stained with crystal violet for 30 min.

For the EdU assay to assess the cell proliferation rate, cells were seeded in 96-well plates and treated as indicated. The ratio of actively proliferating cells was measured using the Cell-Light EdU Apollo488 In Vitro Kit (Ribobio, C10310-3).

### Intracellular ROS content and lipid peroxidation assay

Visualization of intracellular ROS generation was determined using the DCFH-DA assay (Beyotime, S0033S). Cells were seeded onto 6-well-plates at the concentration of 3 × 10^5^/well and incubated overnight. Cells were harvested according to the manufacturer’s instructions. The cells were subjected to different treatment and then incubated for 20 min at 37 ℃ with 10 µM DCFH-DA. The fluorescence intensity was evaluated by the fluorescence microscopy and flow cytometry.

For intracellular lipid peroxidation assay, LoVo, LoVo/CDDP and RKO cells were seeded on sterilized glass coverslips in 24-well culture plates at a density of 2 × 10^5^ cells/mL and allowed to grow overnight. The cells were treated according to the indicated strategy. After two PBS washes, the cells were stained with 10 μm C11-BODIPY 581/591 dye (Thermo Fisher Scientific, D3861) for 15 min at 37 °C in serum-free medium. The cells were rinsed twice with PBS and then fixed for 2 h with 4% paraformaldehyde. Nuclei were stained for 8 min with 0.2 µg/mL DAPI solution (Solarbio, C0060) and rinsed five times with PBS. Images were captured by confocal laser scanning microscopy (CLSM) (Carl Zeiss Micro Imaging 800) and image analysis was performed using ZEN blue software. Cells were cultured into 6-well plates, repeat the above treatments and then collected by trypsinization for flow cytometry analysis.

For intracellular hypoxia assay, the Hypoxia detection kit (Enzo Life Sciences Inc., Farmingdale, USA) was employed. LoVo, LoVo/CDDP and RKO cells were seeded on sterilized glass coverslips in 24-well culture plates at a density of 2 × 10^5^ cells/mL and cultured overnight in a hypoxic incubation incubator. Cells were processed according to the indicated strategy and then treated with the kit reagent mix according to the manufacturer’s instruction. Thirty min later, cells were washed twice with PBS. Images were captured by confocal laser scanning microscopy (Carl Zeiss Micro Imaging 800) and image analysis was performed using ZEN blue software. Cells were cultured into 6-well plates under hypoxia, repeat the above experiments and then collected by trypsinization for flow cytometry analysis.

### Immunoblotting

Cells were washed with ice-cold PBS and then lysed with RIPA buffer (50 mM Tris, 1.0 mM EDTA, 150 mM NaCl, 0.1% SDS, 1% Triton X-100, 1% sodium deoxycholate, 1 mM PMSF). The samples were analyzed by immunoblotting with the indicated antibodies.

### Animal experiments

To evaluate the anti-tumor effect in vivo, BALB/c-Nude mice (6 weeks, 16–22 g each) were purchased from Chengdu YaoKang Biological Technology Co., Ltd. All animal experiments were approved by the Administration Committee of Experimental Animals in Zhejiang Province and the Ethics Committee of Ningbo University [SYXK(Zhejiang)2019-0005]. Primary tumors were obtained by subcutaneously injecting a suspension of 5 × 10^6^ LoVo/CDDP or RKO cells. The mice were randomly divided into four groups (*n* = 5) when tumor volumes reached 100 mm^3^ and injected every other day with the same volume of saline, CDDP (2 mg/kg), L820 (2 mg/kg), C820 (2 mg/kg) via the tail vein. After tail vein injection for 6 h, mice treated with L820 and C820 received 5 min of NIR irradiation (1.0 W/cm^2^). Body weight and tumor volume were recorded every other day. Tumor size was measured with a caliper and tumor volume was calculated according to the following equation: tumor volume (mm^3^) = (length × width^2^)/2. The tumor inhibition rate was measured according to the following formula (average tumor weight in the control group - average tumor weight in the treatment group)/average tumor weight of the control group × 100%). After finalization of the experiments, mice were sacrificed, and tumors and major organs were dissected and weighed to evaluate the therapeutic efficiency of photothermal ablation in different groups.

To establish a pancreatic cancer tumor recurrence model, 5 × 10^6^ LoVo/CDDP cells were subcutaneously injected into the right posterior side of BALB/c nude mice. The tumors were surgically removed when tumor volumes reached 250 mm^3^. After a one-week recovery following surgery, the mice were randomly divided into three groups and treated every two days with saline, CDDP, and C820 NPs. The tumor volumes and body weights were measured every 2 or 3 days.

To assess the tumor-targeting ability of C820 NPs, free IR820, L820, and C820 NPs solutions were injected via the tail vein into tumor-bearing mice. The fluorescence images were captured at time intervals post-injection (1, 2, 4, 6, 8, and 24 h) using a small animal imaging system (Xtreme, Bruker, Germany). Then, the mice were sacrificed, and the major tissues (brain, heart, liver, spleen, lung, and kidney) were collected and imaged.

To visualize the photothermal conversion efficiency of C820 NPs in vivo, thermal images of different groups of mice irradiated (808 nm or 660 nm) continuously for 5 min with a near-infrared laser are measured using an infrared thermographic camera (E60: Compact Infrared Thermal Imaging Camera; FLIR).

### Statistics

For data management and analysis, Microsoft Excel was utilized. Statistical analysis was performed using GraphPad Prism 9 (GraphPad Software, La Jolla, CA, USA). Measured data were expressed as mean and standard deviation. Groups were compared using the two-tailed Student’s t-test or one way ANOVA. The differences were considered significant based on the obtained *p*-values: * <0.05, ** <0.01, *** <0.001.

### Supplementary Information

**Additional file 1: Fig S1.** Synthesis and characterization of C820 NPs. (A) Synthesis illustration of IR820-OH and L780. (B-D) 1H NMR analysis of IR820 (B), IR820-OH (C), L820 (D). (E) *In vitro* release profiles of IR820-OH from C820 NPs in PBS at pH 7.4, pH 6.5 and pH 5.0, respectively. (F-G) Mass spectrum of IR820-OH (F) and L820 (G). (H-J) Photothermal activity of IR820 (H), IR820-OH (I), L820 (J) dispersed in water at various concentrations. **Fig S2.** Cellular uptake and MTT assay of C820 NPs in colorectal cancer cells. (A, C) Fluorescence microscopy images of C820 NPs absorbed by RKO and LoVo at 0, 1, 2, 4, and 6 h. Scale bar: 150 μm. (B, D) Flow cytometry analysis of the time-dependent cellular absorption of C820. (E-G) Cell viability after treatments without laser irradiation. (H) IC50 of drugs in different groups of CDDP/LoVo, LoVo, and RKO cells without laser irradiation. (I) Apoptosis analysis in RKO cells treated with PBS, CDDP, IR820, L820, C820 NPs, and C820 NPs/Hyp by Annexin V-FITC/PI double staining. **Fig S3.** The underlying mechanism of C820 NPs in cancer therapy. (A) Fluorescence microscopy images and (B and C) flow cytometry quantitative analysis for intracellular ROS generation of RKO cells using DCFH-DA as a probe. Scale bar: 150μm. (λ = 808 nm for IR820 and 660nm for L820 and C820 NPs, *P* = 1.0 W/cm^2^; irradiation time = 60s). (D) Confocal laser scanning microscopy (CLSM) images and (E and F) flow cytometry quantitative analysis of the C11-BODIPY (581/591) probe detected lipid peroxidation in RKO cells. Scale bar: 20 μm. (λ = 808 nm for IR820 and 660nm for L820 and C820 NPs, *P* = 1.0 W/cm^2^; irradiation time = 60s). (G) Immunoblot analysis of ferroptosis markers (GPX4) in RKO cells treated as indicated. (H-I) Representative transmission electron microscopy images of C820 NPs-induced ferroptosis in colorectal cancer cells in LoVo cells (H) and RKO cells (I). Scale bar: 1 μm/500 nm. (λ = 808 nm for IR820 and 660nm for L820 and C820 NPs, *P* = 1.0 W/cm2; irradiation time = 60s). (J) CLSM images of LoVo/CDDP cells treated accordingly and stained with hypoxia probes in the presence of 1% O^2^. Scale bar: 30 μm. (K) Statistical analysis of mean fluorescence intensity in LoVo/CDDP cells treated with hypoxia and different drugs. (L) Flow cytometry analysis of intracellular hypoxia level in LoVo/CDDP cells after different treatments in a hypoxic environment. Data represent means ± SD (*n* = 3, one-way ANOVA, **P < 0.01, ***P < 0.001). **Fig S4.** Biosafety of C820 NPs in vivo.  (A) Fluorescence intensity statistics of BALB/c nude mice bearing RKO tumors at different time points after the injection of C820 NPs. (B) Fluorescence intensity statistics of tumors and different organs excised at 24 h post-injection with IR820, L820 or C820. (C) H&E staining of main organs under different treatment. Scale bar: 50 μm. (D-G) Biochemical analysis of the peripheral blood serum after treatment with saline, free CDDP, L820 and C820 NPs with laser irradiation (1 W∙cm^-2^, 5 min), (*n*=5). Data represent means ± SD (*n* = 3, one-way ANOVA, **P < 0.01, ***P < 0.001). **Fig S5.** Anti-tumor properties of C820 NPs in tumor-bearing mice bearing drug-resistant cells. (A-B) Biodistribution and photothermal (C-D) profile of C820 NPs in tumor-bearing BALB/c mouse model. (E) H&E staining of main organs and tumor under different treatment. Scale bar: 50 μm. (F-I) Biochemical analysis of the peripheral blood serum after treatment with saline, free CDDP, L820 and C820 NPs with laser irradiation (1 W∙cm^-2^, 5 min), (*n*=5). Data represent means ± SD (*n* = 3, one-way ANOVA, **P < 0.01, ***P < 0.001). **Fig S6.** Comparison of NOX and SOD Levels in Tumor Tissue with and without Laser Irradiation. A. Photographs of tumors from each group, visually illustrating the differences in tumor growth and response to treatment. (C820+Laser: 660 nm; *P* = 1.0 W/cm^2^) B. Tumor volume change graph. C. Final tumor volumes and the inhibition rate. D. Representative images of immunostaining. Scale bar: 50 μm.

## Data Availability

The datasets used and/or analyzed during the current study are available from the corresponding author on reasonable request.
