# Supplementary material for: Laser-activatable oxygen self-supplying nanoplatform for efficiently overcoming colorectal cancer resistance by enhanced ferroptosis and alleviated hypoxic microenvironment
Source: Biomater Res. 2023 Sep 23;27:92. doi: 10.1186/s40824-023-00427-1 (PMC10518107; doi:10.1186/s40824-023-00427-1)
Supplement: Supplementary file 1 — Additional file 1: Fig S1. Synthesis and characterization of C820 NPs. (A) Synthesis illustration of IR820-OH and L780. (B-D) 1H NMR analysis of IR820 (B), IR820-OH (C), L820 (D). (E) In vitro release profiles of IR820-OH from C820 NPs in PBS at pH 7.4, pH 6.5 and pH 5.0, respectively. (F-G) Mass spectrum of IR820-OH (F) and L820 (G). (H-J) Photothermal activity of IR820 (H), IR820-OH (I), L820 (J) dispersed in water at various concentrations. Fig S2. Cellular uptake and MTT assay of C820 NPs in colorectal cancer cells. (A, C) Fluorescence microscopy images of C820 NPs absorbed by RKO and LoVo at 0, 1, 2, 4, and 6 h. Scale bar: 150 μm. (B, D) Flow cytometry analysis of the time-dependent cellular absorption of C820. (E-G) Cell viability after treatments without laser irradiation. (H) IC50 of drugs in different groups of CDDP/LoVo, LoVo, and RKO cells without laser irradiation. (I) Apoptosis analysis in RKO cells treated with PBS, CDDP, IR820, L820, C820 NPs, and C820 NPs/Hyp by Annexin V-FITC/PI double staining. Fig S3. The underlying mechanism of C820 NPs in cancer therapy. (A) Fluorescence microscopy images and (B and C) flow cytometry quantitative analysis for intracellular ROS generation of RKO cells using DCFH-DA as a probe. Scale bar: 150μm. (λ = 808 nm for IR820 and 660nm for L820 and C820 NPs, P = 1.0 W/cm2; irradiation time = 60s). (D) Confocal laser scanning microscopy (CLSM) images and (E and F) flow cytometry quantitative analysis of the C11-BODIPY (581/591) probe detected lipid peroxidation in RKO cells. Scale bar: 20 μm. (λ = 808 nm for IR820 and 660nm for L820 and C820 NPs, P = 1.0 W/cm2; irradiation time = 60s). (G) Immunoblot analysis of ferroptosis markers (GPX4) in RKO cells treated as indicated. (H-I) Representative transmission electron microscopy images of C820 NPs-induced ferroptosis in colorectal cancer cells in LoVo cells (H) and RKO cells (I). Scale bar: 1 μm/500 nm. (λ = 808 nm for IR820 and 660nm for L820 and C820 NPs, P = 1.0 W/cm2; irra [file 40824_2023_427_MOESM1_ESM.docx]

# Supporting information

# Laser-activatable Oxygen Self-supplying Nanoplatform for Efficiently Overcoming Colorectal Cancer Resistance by Enhanced Ferroptosis and Alleviated Hypoxic Microenvironment

Hao Jiang^a,1^, Hailong Tian^b,1^, Zhihan Wang^b,1^, Bowen Li^b^, Rui Chen^a^, Kangjia Luo^a^, Shuaijun Lu^a^, Edouard C. Nice^c^, Wei Zhang^b^, Canhua Huang^a,b^, Yuping Zhou^a,^ *, Shaojiang Zheng^d,^ *, Feng Gao^a,^ *

*^a^**The First Hospital of Ningbo University, Ningbo, 315020, China.*

*^b^**State Key Laboratory of Biotherapy and Cancer Center, West China Hospital, West China School of Basic Medical Sciences & Forensic Medicine, Sichuan University, and Collaborative Innovation Center for Biotherapy, Chengdu, 610041, China.*

*^c^**Department of Biochemistry and Molecular Biology, Monash University, Clayton, VIC, 3800, Australia.*

*^d^Hainan Cancer Center and Tumor Institute, The First Affiliated Hospital of Hainan Medical University, Haikou 570102, China.*

*^e^Key Laboratory of Tropical Cardiovascular Diseases Research of Hainan Province,Hainan Women and Children Medical Center, Key Laboratory of Emergency and Trauma of Ministry of Education, Hainan Medical University, Haikou 571199, China.*

*^1^ These authors contributed equally to this work.*

^*^ *Corresponding authors.*

#
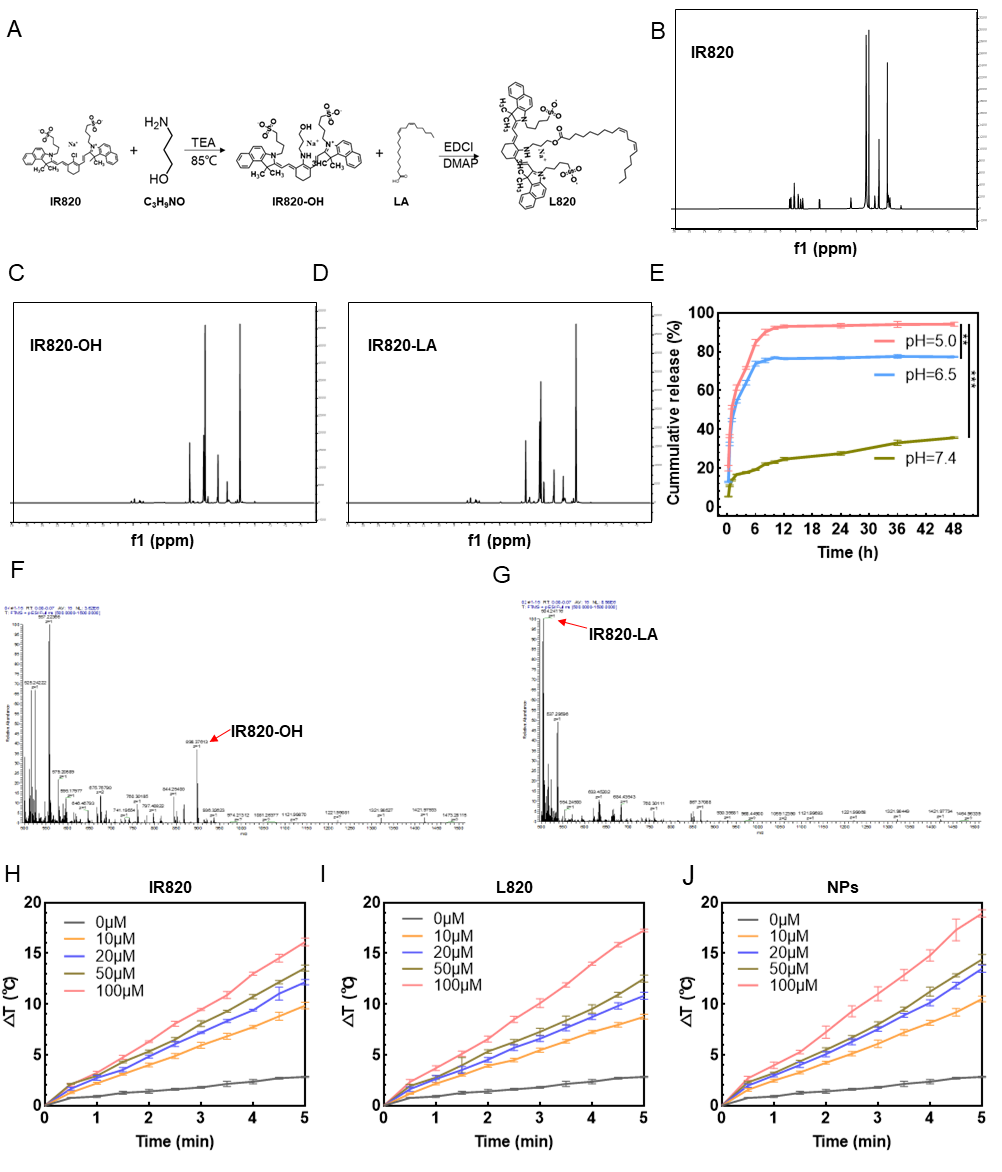


**Fig. S1. Synthesis and characterization of C820 NPs.** (A) Synthesis illustration of IR820-OH and L780. (B-D) 1H NMR analysis of IR820 (B), IR820-OH (C), L820 (D). (E) *In vitro* release profiles of IR820-OH from C820 NPs in PBS at pH 7.4, pH 6.5 and pH 5.0, respectively. (F-G) Mass spectrum of IR820-OH (F) and L820 (G). (H-J) Photothermal activity of IR820 (H), IR820-OH (I), L820 (J) dispersed in water at various concentrations.


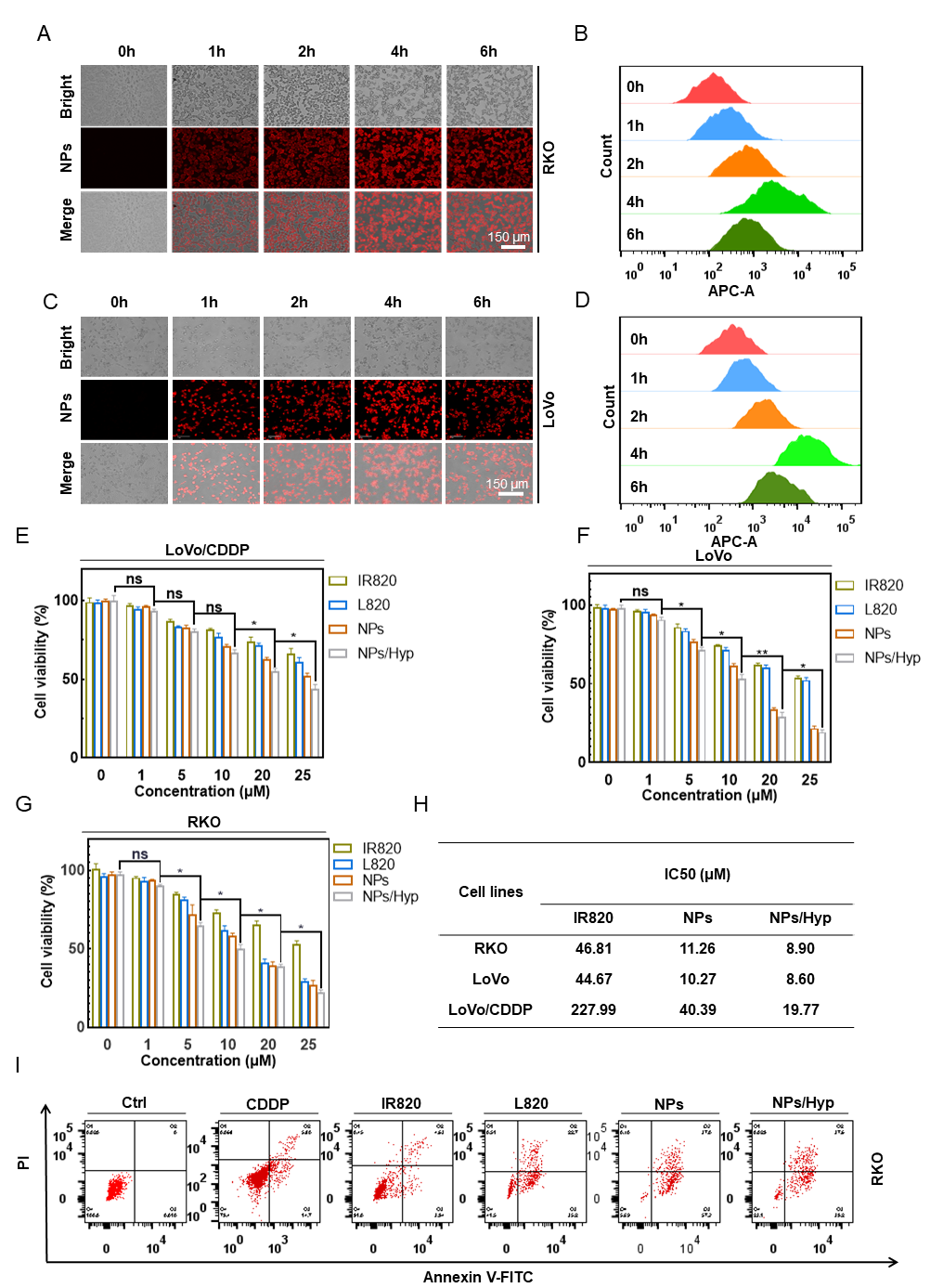


**Fig. S2. Cellular uptake and MTT assay of C820 NPs in colorectal cancer cells.** (A, C) Fluorescence microscopy images of C820 NPs absorbed by RKO and LoVo at 0, 1, 2, 4, and 6 h. Scale bar: 150 μm. (B, D) Flow cytometry analysis of the time-dependent cellular absorption of C820. (E-G) Cell viability after treatments without laser irradiation. (H) IC50 of drugs in different groups of CDDP/LoVo, LoVo, and RKO cells without laser irradiation. (I) Apoptosis analysis in RKO cells treated with PBS, CDDP, IR820, L820, C820 NPs, and C820 NPs/Hyp by Annexin V-FITC/PI double staining.

**
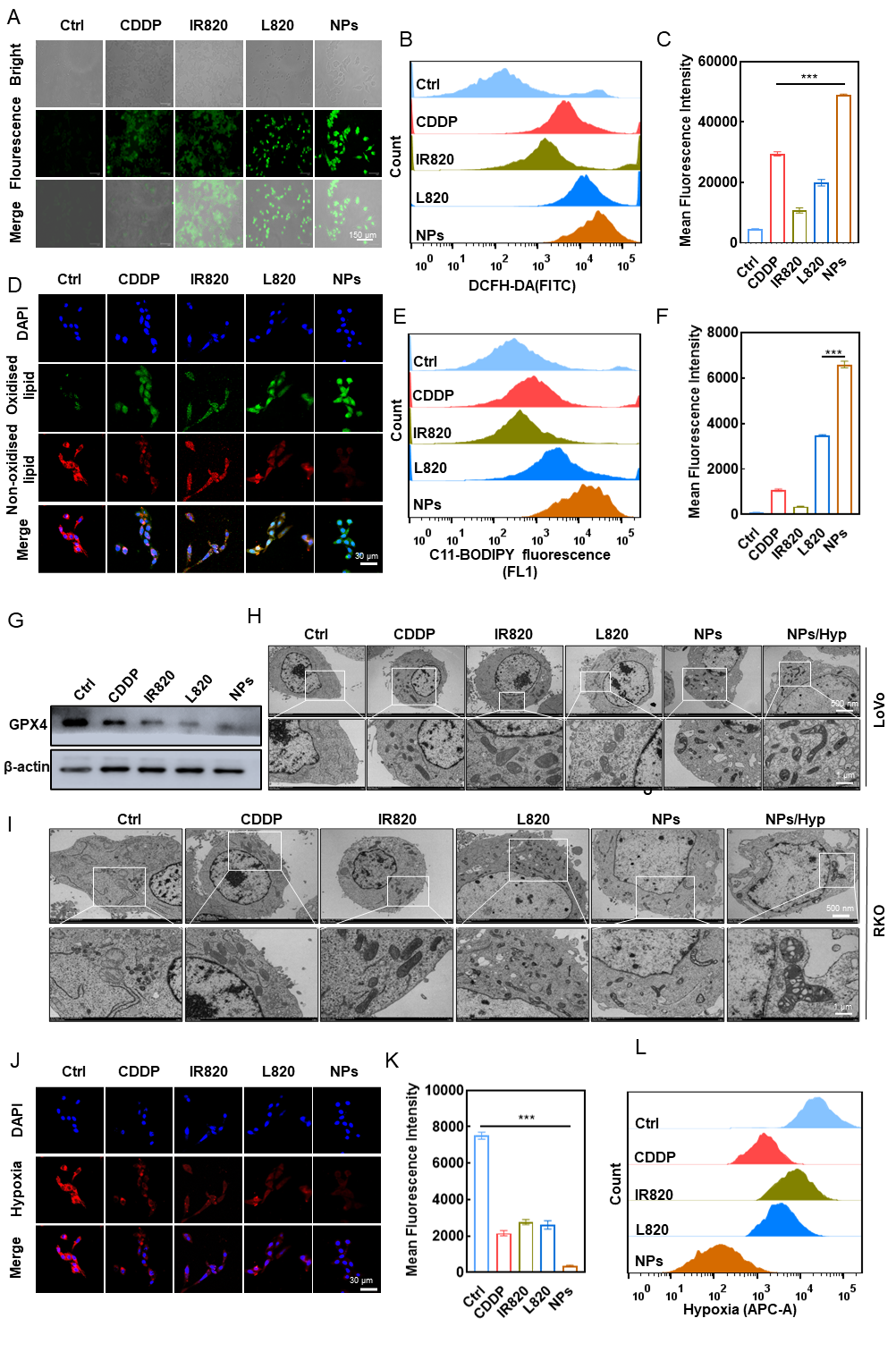
Fig. S3. The underlying mechanism of C820 NPs in cancer therapy.** (A) Fluorescence microscopy images and (B and C) flow cytometry quantitative analysis for intracellular ROS generation of RKO cells using DCFH-DA as a probe. Scale bar: 150μm. (λ = 808 nm for IR820 and 660nm for L820 and C820 NPs, P = 1.0 W/cm^2^; irradiation time = 60s). (D) Confocal laser scanning microscopy（CLSM）images and (E and F) flow cytometry quantitative analysis of the C11-BODIPY (581/591) probe detected lipid peroxidation in RKO cells. Scale bar: 20 μm. (λ = 808 nm for IR820 and 660nm for L820 and C820 NPs, P = 1.0 W/cm^2^; irradiation time = 60s). (G) Immunoblot analysis of ferroptosis markers (GPX4) in RKO cells treated as indicated. (H-I) Representative transmission electron microscopy images of C820 NPs-induced ferroptosis in colorectal cancer cells in LoVo cells (H) and RKO cells (I). Scale bar: 1 μm/500 nm. (λ = 808 nm for IR820 and 660nm for L820 and C820 NPs, P = 1.0 W/cm^2^; irradiation time = 60s). (J) CLSM images of LoVo/CDDP cells treated accordingly and stained with hypoxia probes in the presence of 1% O_2_. Scale bar: 30 μm. (K) Statistical analysis of mean fluorescence intensity in LoVo/CDDP cells treated with hypoxia and different drugs. (L) Flow cytometry analysis of intracellular hypoxia level in LoVo/CDDP cells after different treatments in a hypoxic environment. Data represent means ± SD (n = 3, one-way ANOVA, ***P* < 0.01, ****P* < 0.001).

**
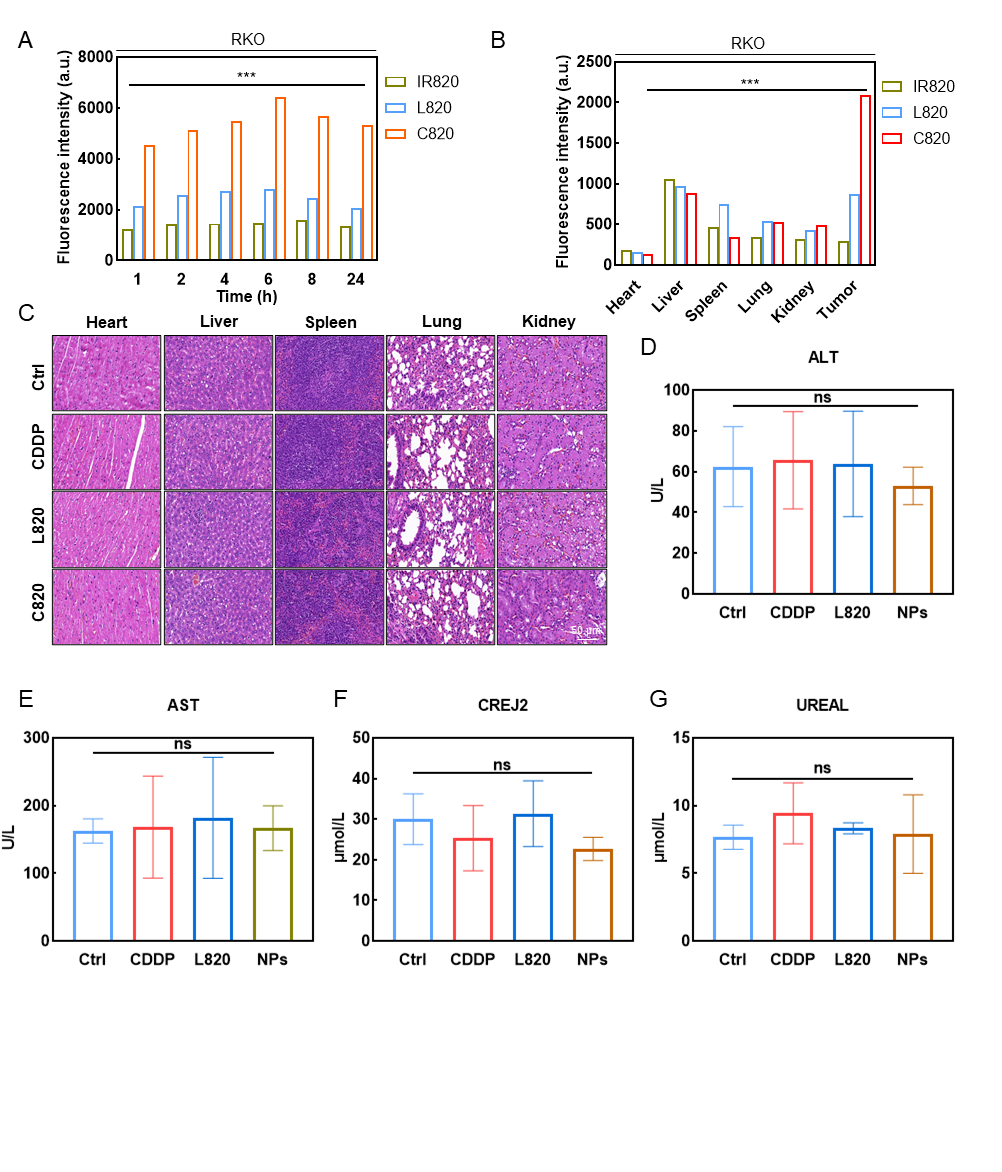
Fig. S4. Biosafety of C820 NPs in vivo.** (A) Fluorescence intensity statistics of BALB/c nude mice bearing RKO tumors at different time points after the injection of C820 NPs. (B) Fluorescence intensity statistics of tumors and different organs excised at 24 h post-injection with IR820, L820 or C820. (C) H&E staining of main organs under different treatment. Scale bar: 50 μm. (D-G) Biochemical analysis of the peripheral blood serum after treatment with saline, free CDDP, L820 and C820 NPs with laser irradiation (1 W/cm^2^, 5 min), (n=5). Data represent means ± SD (n = 3, one-way ANOVA, ***P* < 0.01, ****P* < 0.001).

**
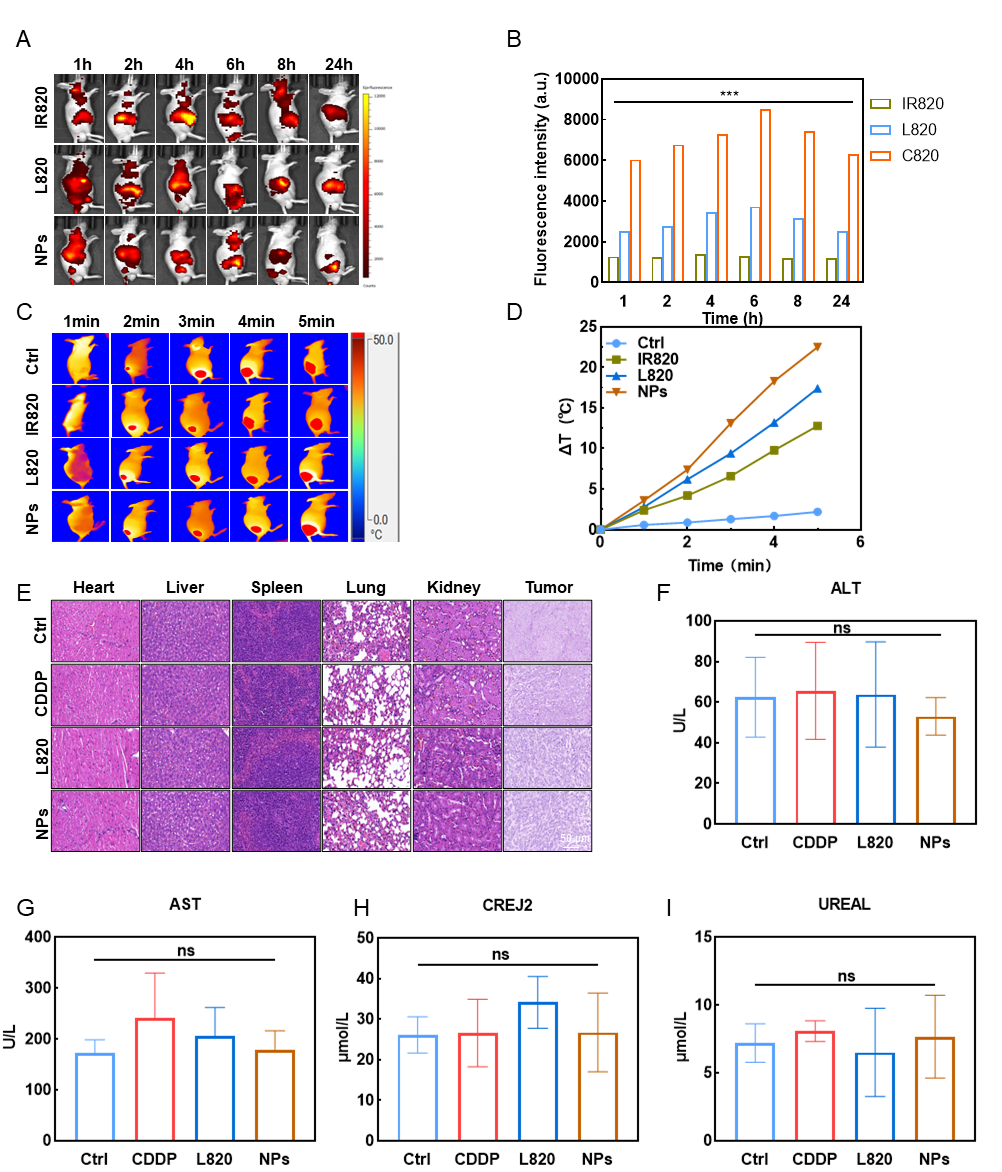
**

**Fig. S5. Anti-tumor properties of C820 NPs in tumor-bearing mice bearing drug-resistant cells.** (A-B) Biodistribution and photothermal (C-D) profile of C820 NPs in tumor-bearing BALB/c mouse model. (E) H&E staining of main organs and tumor under different treatment. Scale bar: 50 μm. (F-I) Biochemical analysis of the peripheral blood serum after treatment with saline, free CDDP, L820 and C820 NPs with laser irradiation (1 W/cm^2^, 5 min), (n=5). Data represent means ± SD (n = 3, one-way ANOVA, ***P* < 0.01, ****P* < 0.001).


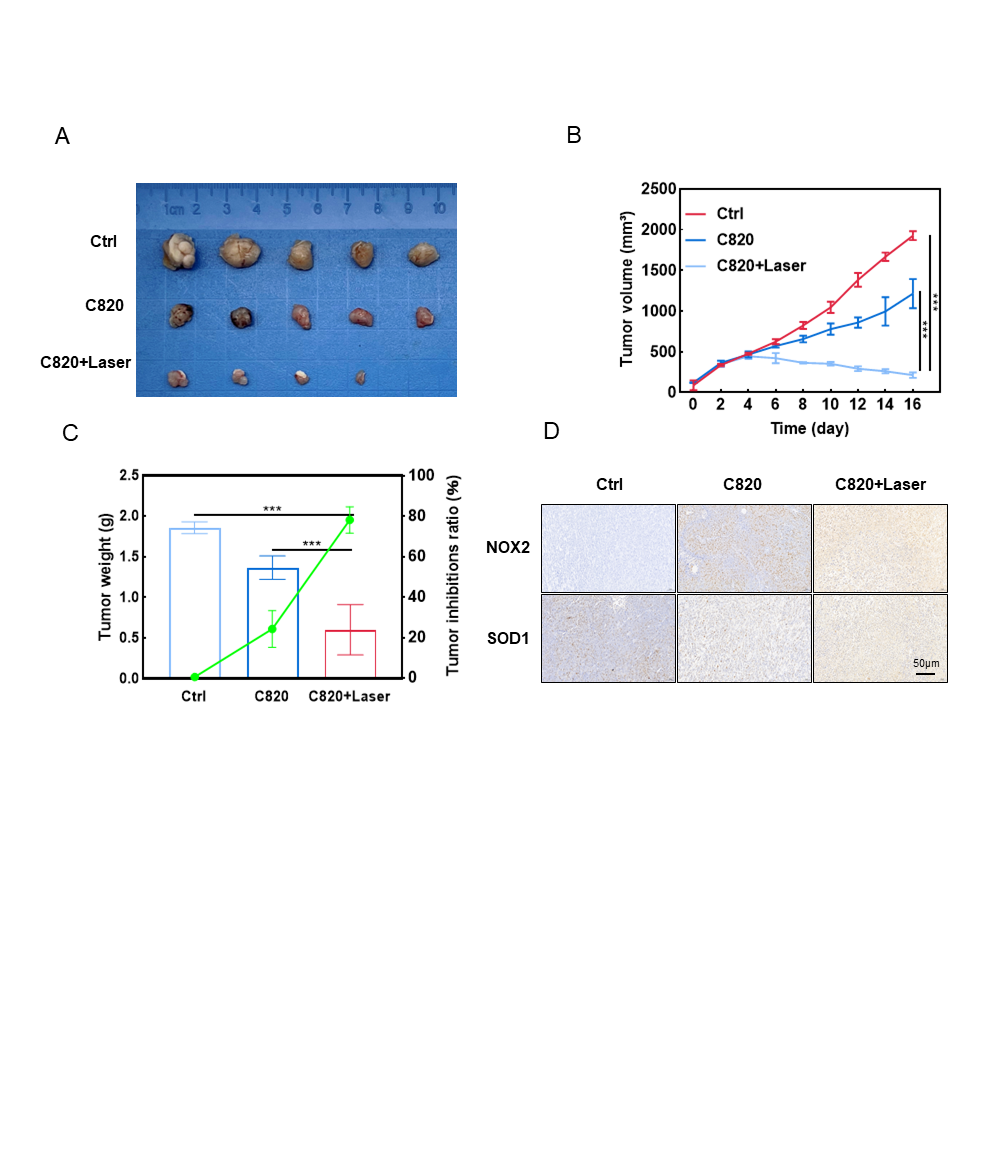


**Fig. S6.** **Comparison of NOX and SOD Levels in Tumor Tissue with and without Laser Irradiation.** A. Photographs of tumors from each group, visually illustrating the differences in tumor growth and response to treatment. (C820+Laser: 660 nm; P = 1.0 W/cm^2^) B. Tumor volume change graph. C. Final tumor volumes and the inhibition rate. D. Representative images of immunostaining. Scale bar: 50 μm.
